# *Bona fide* atypical scrapie faithfully reproduced for the first time in a rodent model

**DOI:** 10.1186/s40478-022-01477-7

**Published:** 2022-12-13

**Authors:** Enric Vidal, Manuel A. Sánchez-Martín, Hasier Eraña, Sonia Pérez Lázaro, Miguel A. Pérez-Castro, Alicia Otero, Jorge M. Charco, Belén Marín, Rafael López-Moreno, Carlos M. Díaz-Domínguez, Mariví Geijo, Montserrat Ordóñez, Guillermo Cantero, Michele di Bari, Nuria L. Lorenzo, Laura Pirisinu, Claudia d’Agostino, Juan María Torres, Vincent Béringue, Glenn Telling, Juan J. Badiola, Martí Pumarola, Rosa Bolea, Romolo Nonno, Jesús R. Requena, Joaquín Castilla

**Affiliations:** 1 Unitat Mixta d’Investigació IRTA-UAB en Sanitat Animal, Centre de Recerca en Sanitat Animal (CReSA), Campus de la Universitat Autònoma de Barcelona (UAB), Bellaterra, Catalonia Spain; 2 IRTA Programa de Sanitat Animal, Centre de Recerca en Sanitat Animal (CReSA), Campus de la Universitat Autònoma de Barcelona (UAB), Bellaterra, Catalonia Spain; 3 Transgenic Facility. Department of Medicine, University of Salamanca, 37007 Salamanca, Spain; 4 Centro de Investigación Cooperativa en Biociencias (CIC BioGUNE), Laboratorio de Investigación de Priones, Basque Research and Technology Alliance (BRTA), Derio, Bizkaia Spain; 5 ATLAS Molecular Pharma S. L., Derio, Bizkaia Spain; 6 Centro de Investigación Biomédica en Red de Enfermedades Infecciosas (CIBERINFEC), Instituto de Salud Carlos III, Madrid, Spain; 7 Centro de Encefalopatías y Enfermedades Transmisibles Emergentes, Facultad de Veterinaria, Universidad de Zaragoza–IA2, Zaragoza, Spain; 8 Animal Health Department, NEIKER-Basque Institute for Agricultural Research and Development, Basque Research and Technology Alliance (BRTA), Derio, Spain; 9 Department of Food Safety, Nutrition and Veterinary Public Health, Istituto Superiore Di Sanità, 00161 Rome, Italy; 10 CIMUS Biomedical Research Institute, University of Santiago de Compostela-IDIS, Santiago, Spain; 11 Centro de Investigación en Sanidad Animal (CISA), Centro Superior de Investigaciones Científicas (CSIC) Valdeolmos, Instituto Nacional de Investigación y Tecnología Agraria y Alimentaria (INIA), 28130 Madrid, Spain; 12 Molecular Virology and Immunology, Institut National de La Recherche Agronomique (INRA), Université Paris-Saclay, Jouy-en-Josas, France; 13 Prion Research Center (PRC) and the Department of Microbiology, Immunology, and Pathology, Colorado State University, Fort Collins, CO USA; 14 Departament de Medicina i Cirurgia Animals, Facultat de Veterinària, Campus de UAB, Bellaterra, 08193 Barcelona, Catalonia Spain; 15 IKERBASQUE, Basque Foundation for Science, Bilbao, Bizkaia Spain

**Keywords:** Atypical, Scrapie, Spontaneous, Prion disease, Isoleucine

## Abstract

Atypical Scrapie, which is not linked to epidemics, is assumed to be an idiopathic spontaneous prion disease in small ruminants. Therefore, its occurrence is unlikely to be controlled through selective breeding or other strategies as it is done for classical scrapie outbreaks. Its spontaneous nature and its sporadic incidence worldwide is reminiscent of the incidence of idiopathic spontaneous prion diseases in humans, which account for more than 85% of the cases in humans. Hence, developing animal models that consistently reproduce this phenomenon of spontaneous PrP misfolding, is of importance to study the pathobiology of idiopathic spontaneous prion disorders. Transgenic mice overexpressing sheep PrP^C^ with I112 polymorphism (TgShI112, 1–2 × PrP levels compared to sheep brain) manifest clinical signs of a spongiform encephalopathy spontaneously as early as 380 days of age. The brains of these animals show the neuropathological hallmarks of prion disease and biochemical analyses of the misfolded prion protein show a ladder-like PrP^res^ pattern with a predominant 7–10 kDa band. Brain homogenates from spontaneously diseased transgenic mice were inoculated in several models to assess their transmissibility and characterize the prion strain generated: TgShI112 (ovine I112 ARQ PrP^C^), Tg338 (ovine VRQ PrP^C^), Tg501 (ovine ARQ PrP^C^), Tg340 (human M129 PrP^C^), Tg361 (human V129 PrP^C^), TgVole (bank vole I109 PrP^C^), bank vole (I109I PrP^C^), and sheep (AHQ/ARR and AHQ/AHQ churra-tensina breeds). Our analysis of the results of these bioassays concludes that the strain generated in this model is indistinguishable to that causing atypical scrapie (Nor98). Thus, we present the first faithful model for a *bona fide*, transmissible, ovine, atypical scrapie prion disease.

**Supplementary Information:**

The online version contains supplementary material available at 10.1186/s40478-022-01477-7.

## Introduction

Transmissible spongiform encephalopathies (TSE) or prion diseases are a group of transmissible neurodegenerative diseases associated with the misfolding of the endogenous cellular prion protein (PrP^C^) into a pathogenic isoform, termed PrP^res^, which is partially resistant to proteases and induces neurotoxicity [64]. This misfolding event can be spontaneous, either idiopathic (without a known cause), linked with a mutation in the gene encoding PrP^C^ (*PRNP*), or acquired through exposure to external sources of misfolded prions [9, 15, 16, 31, 38, 59].

Acquired animal prion diseases, such as scrapie in small ruminants or chronic wasting disease (CWD) in cervids, can be transmitted horizontally with high efficiency, while others, such as BSE, rely on human interventions (recirculation of meat and bone meal contaminated with prions, for instance) [77, 79]. Over the years, several supposedly idiopathic spontaneous prion diseases have also been described in animals. These are known as atypical BSE cases in cattle, namely BSE-L [18] and BSE-H [12], and the atypical scrapie or Nor98-like cases in small ruminants [11]. In the case of BSE-L, a clear potential for zoonotic risk has been established [48, 81]. However, in contrast to human prion diseases, no genetic cases have been described in animals to date.

Nor98 cases are of particular interest since they have been linked to the emergence of the BSE agent when inoculated in a host with bovine [41] or porcine PrP^C^ [27, 49]. The cause of Nor98 cases has not been yet established, and although some cases are associated with specific polymorphisms in the *PNRP* gene, such as the presence of phenylalanine in codon 141 [53] or the presence of histidine in codon 154 [21], these polymorphisms do not explain all cases. Thus, an idiopathic spontaneous origin is the current hypothesis. Atypical/Nor98 scrapie is a worldwide distributed disease occurring in small ruminants even in classical scrapie-free regions such as Australia [22]. Thus, despite the apparently low horizontal transmission capacity of these atypical prions, which do not seem to be related to outbreaks unlike those that cause classical scrapie, their spontaneous occurrence remains a concern given their potential transmissibility and potential alterations upon transmission.

Apart from their origin and horizontal transmission ability, there are several other differences between classical and atypical scrapie regarding clinical, neuropathological, and biochemical features. For instance, instead of showing the classical three-banded pattern on Western blotting upon proteinase K (PK) digestion, corresponding to the three 27–30 kDa PrP^res^ glycoforms, Nor98-associated PrP^res^ is characterized by an enhanced PK sensitivity and a multi-banded or ladder-like pattern with a predominant band of 7–14 kDa. A similar atypical biochemical signature, which reflects a different structural arrangement [5], has also been described in other human disorders of idiopathic or genetic origin [32, 55, 63, 67].

Although animal models and, most importantly, transgenic mouse models have been essential to study acquired prion disorders through experimental inoculations, their ability to model genetic or spontaneous idiopathic prion disorders is more limited. Mimicking the main event underlying these diseases, which consists of the putatively spontaneous misfolding of PrP^C^ into PrP^res^, is hindered by the low frequency of such phenomenon [9, 59], as reflected by the low incidence of spontaneous idiopathic prion diseases and the difficulties of reproducing genetic diseases in animals through introduction of human *PRNP* mutations. Nonetheless, a few models have succeeded in reproducing genetic mutation-based prionopathies. These include the ki-3F4-FFI mice that harbour the D177N mutation, equivalent to the human mutation responsible for FFI [43] and the D178N Tg(FFI) also reproducing FFI phenotype [14], the Tg(A116V) mice mimicking A117V GSS [84], the TgMHuME199K mimicking E200K genetic CJD [30], the 113LBoPrP-Tg mice that incorporated the P113L substitution, homologous to the P102L mutation causing GSS in humans in a bovine *PRNP* transgenic mouse model [70], the P102L mutation of GSS in the murine prnp [68] or the 117VVTg30 mice also mimicking A117V GSS [6]. Despite the fact that all these models succumb to a spontaneous neurological illness related to misfolded PrP accumulation, these misfolded products are poorly infectious, if infectious at all in wild type animals, where they show incomplete attack rates or require multiple serial inoculations, raising doubts on their infectious or transmissible nature or indicating that homotypic mutant PrP expression is required for propagation.

Moreover, in spite of the relative success of the previous models in mimicking genetic prion diseases, the study of spontaneous idiopathic prion disease in animal models, which occurs in absence of mutations in the *PRNP* gene, is more challenging. The most successful approach to date explores the particular susceptibility of bank voles (*Myodes glareolus*) to most prion strains [1, 7, 54, 75] by producing transgenic mouse models with the bank vole PrP^C^. These models, particularly when PrP^C^ is overexpressed, spontaneously develope a *bona fide* transmissible prion disease that is determined by the presence of an isoleucine in position 109 [56, 57, 76]. The other naturally occurring polymorphism at that position, M109, has not been linked to spontaneity [17, 76]. Other authors however, demonstrated that the M109 polymorph, when overexpressed, was also associated to PrP^res^ deposition, although this PrP^res^ was not infectious, and proposed other polymorphisms of the bank vole *PRNP* as responsible for its enhanced susceptibility and tendency towards spontaneous misfolding [44].

Interestingly, the presence of isoleucine in this position is a naturally occurring polymorphism not only in bank vole, but in at least 10 mammal species including cattle [86], horse [87], skunk [88] and also in sheep, described in the Tibetan breed [85]. Albeit to date no cases of spontaneous prion disease are reported in animals with this polymorphism.

Due to the lack of animal models that mimic a spontaneous prionopathy in domestic animals, our goal was to explore whether the equivalent polymorphism in sheep (i.e. I112) would yield an ovine PrP^C^ more prone to spontaneous misfolding in a transgenic mouse model.

As predicted, our mouse model TgShI112 spontaneously developed a prion disease that, interestingly, presented strain features indistinguishable to that of atypical (Nor98) scrapie, providing the first animal model of clearly transmissible and atypical prion disease in absence of pathology-associated mutations.

## Materials and methods

### Generation of TgShI112 mouse lines 460 and 445

After isolation by PCR amplification from genomic DNA, extracted using GeneJET™ Genomic DNA Purification Kit (Fermentas) from a sheep brain tissue sample using 5’ CCGGAATTCCGGCGTACGATGGTGAAAAGCCACATAGGC 3’ and 5’ CTAGTCTAGACTAGGCCGGCCCTATCCTACTATGAGAAAAATG 3’ as primers, the open reading frame (ORF) of the sheep *PRNP* gene with A136, R154 and Q171 polymorphisms was cloned into the pDrive vector (Qiagen), being the sequence identical to that of the Genbank entry NP_001009481.1. The genetic construct containing the sheep M112I substitution was carried out by two-step PCR site-directed mutagenesis using the pDrive sheep construct as template, using primers 5’ CCGGAATTCCGGCGTACGATGGTGAAAAGCCACATAGGC 3’ with 5’ GCTCCTGCCACATGCTTAATGTTGGTTTTTGG 3’ and 5’ CTAGTCTAGACTAGGCCGGCCCTATCCTACTATGAGAAAAATG 3’ with 5’ CCAAAAACCAACATTAAGCATGTGGCAGGAGC 3’. Then using the previous fragments as templates and primers 5’ CCGGAATTCCGGCGTACGATGGTGAAAAGCCACATAGGC 3’ and 5’ CTAGTCTAGACTAGGCCGGCCCTATCCTACTATGAGAAAAAT 3’, the sheep M112I-PrP ORF was generated and cloned into the pDrive vector.The ORF from sheep *PRNP* M112I was excised from the cloning vector by using the restriction enzymes BsiWI (Thermo Fisher Scientific Inc.) and FseI (New England Biolabs Ltd.) and then inserted into a modified version of MoPrP.Xho vector [13] as described previously [19], which was also digested with BstWI and FseI. This vector contains the murine PrP promoter and exon-1, intron-1, exon-2 and 3’ untranslated sequences. Finally, the construct was excised using NotI and purified with an Invisorb Spin DNA Extraction Kit (Inviteck) according to the manufacturer recommendations.

Transgenic mouse founders were generated by microinjection of NotI excised DNA into pronuclei following standard procedures [20]. DNA extracted from tail biopsies was analyzed by PCR using specific primers for the mouse exon 2 and 3’ untranslated sequences (5’ GAACTGAACCATTTCAACCGAG 3’ and 5’ AGAGCTACAGGTGGATAACC 3’). Those which tested positive were bred to mice null for the mouse *Prnp* gene in order to avoid endogenous expression of mouse prion protein. Absence of the mouse endogenous *Prnp* was assessed using the following primers: 5’ ATGGCGAACCTTGGCTACTGGC 3’ and 5’ GATTATGGGTACCCCCTCCTTGG 3’. The sheep PrP expression levels of brain homogenates from transgenic mouse founders were determined by Western blot using anti-PrP mAb D18 [46] and compared with the PrP expression levels from sheep brain homogenates.

The international code to identify these transgenic mouse lines are 129OLA-Prnptm2Edin-Tg(sheepPrnp)1/Sal and 129OLA-Prnptm2Edin-Tg(sheepPrnp)2/Sal although throughout the article they are referred to as TgShI112 (L460) and TgShI112 (L456) mice, respectively. The two lines have slightly different PrP^C^ expression levels (Additional file 1: Fig. S1).

### Preparation of inocula

10% brain homogenates (w/V) from sheep diagnosed of atypical scrapie in the PRIOCAT laboratory, IRTA-CReSA (Barcelona, Catalonia), sheep infected with SSBP/1 (TSE Resource Centre, University of Edinburgh) and BSE infected sheep (SheepBSE, kindly provided by Dr.Olivier Andreoletti, UMR INRAE-ENVT, Toulouse) were prepared manually using a glazed mortar and pestle in Phosphate buffered saline (PBS, Fisher Reagents) with Complete Protease inhibitor cocktail (Roche) and further diluted to 1% in Dulbecco’s PBS (DPBS, Gibco) for direct intracerebral inoculation.

An inoculum was prepared by pooling the 10% homogenates of five TgShI112 mouse brains with a confirmed disease phenotype, which was also further diluted to 1% in DPBS for direct intracerebral inoculation, this inoculum was named ShTgSPON.

### Bioassays with ShTgSPON, atypical and classical scrapie inocula in transgenic mice and bank voles

Mice of 42–56 days of age were intracerebrally inoculated under gaseous anesthesia (Isoflurane) through the right parietal bone. A 50 µl SGC precision syringe was used with a 25 G gauge needle to inoculate a final volume of 20 µl per animal. A dose of buprenorphine was subcutaneously injected before recovery to consciousness to reduce post-inoculation pain. Mice were kept in a controlled environment at a room temperature of 22 ºC, 12 h light-darkness cycle and 60% relative humidity in HEPA filtered cages (both air inflow and extraction) in ventilated racks. The mice were fed ad libitum, observed daily and their clinical status assessed twice a week. The presence of TSE-associated clinical signs was scored (0–3) including: cyphosis, gait abnormalities, altered coat state, depressed mental state, flattened back, eye discharge, hyperactivity, loss of body condition and incontinence. Positive TSE diagnosis relied principally on the detection of PrP^res^ (by either immunohistochemistry and/or Western blotting, or ELISA) and associated spongiform changes on stained histological sections of the brain parenchyma.

The transgenic mouse models used in this paper include Tg338 [23], Tg501 [2], TgVole (1x) [25, 29], TgVole (4x) [56, 57], TgHu340(Met129) and TgHu361(Val 129) [58].

For the bank vole inoculations, brain tissues from TSE affected animals were homogenized at 10% (w/v) in phosphate buffered saline (PBS) and stored at -80 °C. Groups of six-to-eight week old bank voles carrying isoleucine at PRNP codon 109 (Bv109I) were inoculated intracerebrally with 20 μl of homogenate into the left cerebral hemisphere, under ketamine anaesthesia (ketamine 0.1 μg/g). All voles were individually identified by a passive integrated transponder. Animals were examined twice a week until neurological signs appeared, after which they were examined daily. The attack rate was calculated as the number of animals scoring positive at post-mortem/number inoculated. Animals found dead or culled for intercurrent disease before 200 days post inoculation and scoring negative at postmortem were excluded from analyses. The survival time for animals scoring positive at post-mortem was calculated as the time from inoculation to culling or death.

### Sample processing and general procedures

When the clinical end-point criteria were reached (quantitatively a clinical score higher than 10, qualitatively an unresponsive mental state or the presence of invalidating motor disturbances), mice were euthanized by decapitation under gaseous anesthesia (Isofluorane). The brain was extracted immediately, divided longitudinally, and placed into 10% phosphate buffered formalin. Transversal sections of the brain were performed at the levels of the medulla oblongata, piriform cortex and optic chiasm. Samples were embedded in paraffin-wax after dehydration through increasing alcohol concentrations and xylene. Four-micrometre sections were mounted on glass microscope slides and stained with hematoxylin and eosin for morphological evaluation. Additional sections were mounted in 3-trietoxysilil-propilamine-coated glass microscope slides for immunohistochemistry. One half of the brain was separated prior to fixation and kept frozen for biochemical analysis.

For the experiments in bank voles, animals were culled with carbon dioxide before neurological impairment was such as to compromise their welfare, in particular their ability to drink and feed adequately. At post-mortem, brains from inoculated voles were removed and divided into two parts by a sagittal paramedian cut. The smaller portion (left part) was frozen for WB analysis of PrP^Sc^ and the larger one (right part) was fixed in formol-saline for histological and PET-blot analyses.

### Sheep bioassay

ShTgSPON prions were intracerebrally inoculated into four sheep of the churra-tensina breed, 2 of them expressing the AHQ/AHQ and another 2 expressing the AHQ/ARR *PRNP* genotype. To check whether the ShTgSPON inoculum reproduced the features of atypical scrapie, one AHQ/AHQ sheep was intracerebrally inoculated with an atypical scrapie/Nor98 isolate. The intracerebral inoculation was performed as described previously[37]. Briefly, a small perforation was made in the cranial bone with the aid of a special bone drill. The place of election was at the level of the frontal sinus one centimetre lateral from the midline. Subsequently, 0.5 mL of inoculum consisting of a 10% homogenate of the ShTgSPON or atypical scarpie agent in sterile saline solution was inoculated in the frontal cortex using a 25G diameter hypodermic needle. This procedure was performed under a deep anaesthetic plane and subsequently to the inoculation, antibiotic therapy was administered.

### ELISA

Frozen mouse brains were thawed and homogenised 1:10 (weight volume) in sterile phosphate buffered saline. They were routinely tested by ELISA (IDEXX, Herdcheck) ultrashort protocol. It is a commercial ELISA based on the affinity of misfolded prions to an anionic substrate (termed Seprion®). A new threshold was defined to adapt to the higher densitometry readings obtained when working with samples with PrP^C^ overexpression: only samples with a ratio spectrophotometry reading/cut-off over 5 were considered positive.

### Immunohistochemistry

Immunohistochemistry (IHC) for detection of PrP^res^ was performed as described previously [66]. Briefly, deparaffinized sections were subjected to epitope unmasking treatments: immersed in formic acid and boiled at low pH (6.15) in a pressure cooker and pre-treated with proteinase K (4 µg/mL). Endogenous peroxidases were blocked by immersion in a 3% H_2_O_2_ in methanol solution. Sections were then incubated overnight with anti-PrP MAb 2G11 primary antibody (1:100, Bio-Rad) for sheep PrP^C^ models and 6C2 (1:1000, CVI-Wageningen UR) for Bank vole PrP^C^ models and 6H4 (1:100, Thermofisher) for human PrP^C^ models, and subsequently visualized using the Goat anti-mouse EnVision system (DAKO) and 3,3’-diaminobenzidine (DAKO) as the chromogen substrate. As a background control, incubation with the primary antibody was omitted.

PrP^res^ immunodetection in sheep was performed as described previously [52]. Sheep brain sections were treated in 98% formic acid for 15 min followed by incubation with proteinase K (4 µg/ml, Roche, Switzerland) for 15 min at 37 ˚C and hydrated autoclaving in citrate buffer. Immunostaining was done in an automated autostainer (DAKO). After blocking endogenous peroxidase (ready-to-use solution, DAKO) immunodetection was performed using the 8G8 antibody (1:200, 1 h at RT; Cayman Chemical). Goat anti-mouse EnVision system (DAKO) was used as the visualization system and diaminobenzidine (DAKO) as the chromogen. Sections were counterstained with hematoxylin.

For bank vole studies histology and PET-blot analyses were performed on formalin-fixed tissues as previously described [54]. Briefly, coronal brain sections were obtained from four antero-posterior levels including the following: (1) telencephalon at midlevel of caudate nucleus, (2) diencephalon at midlevel of thalamus, (3) midbrain, and (4) hindbrain at midlevel of medulla and cerebellum. Sections of 5 µm from the above levels were prepared and stained either with hematoxylin and eosin to assess spongiosis or were subjected to PET-blot using the 6C2 mAb (epitope on bank vole PrP 111–116). Neuropathological assessment was performed on sections stained with hematoxylin and eosin, and lesion profiles were constructed scoring the vacuolar degeneration in nine gray-matter areas of the brain. Vacuolation scores were derived from at least five individual animals per group and are reported as means ± SEM.

### Semi-quantification and data analysis

Mouse histological lesions (i.e. spongiform change) and PrP^res^ immunolabeling were evaluated under a light microscope by a single pathologist. A semi-quantitative approach was used as previously described [72]. Spongiform lesion and PrP^res^ immunolabeling were separately scored. Fourteen different brain regions were chosen: piriform cortex (Pfc), hippocampus (H), occipital cortex (Oc), temporal cortex (Tc), parietal cortex (Pc), frontal cortex (Fc), striatum (S), thalamus (T), hypothalamus (HT), mesencephalon (M), medulla oblongata (Mobl), cerebellar nuclei (Cm), cerebellar vermis (Cv) and cerebellar cortex (Cc). Scores ranging from (0) absence of spongiosis or immunolabeling: (1) mild, (2) moderate, (3) intense and (4) maximum intensity of lesion or immunolabeling were assigned to each brain area studied. Each area was investigated globally as region for the scoring. Brain profiles were plotted as a function of the anatomical areas which were ordered along the horizontal axis to represent the caudo-rostral axis of the encephalon. Graphs were plotted using Microsoft Office Excel software.

### Proteinase K digestion and western blotting

Two different PK digestion procedures were applied to the mouse and sheep brain homogenate samples in order to evaluate the presence of PrP^res^ with a classical three-banded pattern or with an atypical ladder-like multi-banded pattern by Western blotting. To detect PrP^res^ with a classical biochemical signature, applied for the digestion of SSBP/1 classical scrapie isolate used as controls in the Western blot gels, the brain previously homogenized at 10% (w/V) in Phosphate buffered saline (PBS) (Fisher Bioreagents) with Protease inhibitor cocktail (Roche), was mixed with digestion buffer [2% (w/V Tween-20 (Sigma-Aldrich), 2% (V/V) NP-40 (Sigma-Aldrich) and 5% (w/V) Sarkosyl (Sigma-Aldrich) in PBS] at 1:1 (V/V). Proteinase K (Roche) was added to reach a final concentration of 85 µg/ml to each sample and these were incubated at 42 °C for 1 h with moderate shaking. Digestion was stopped by adding loading buffer (NuPage 4X Loading Buffer, Invitrogen) 1:3 (V/V). For the detection of PrP^res^ with atypical biochemical signature, which allows also detection of classical PrP^res^ banding pattern, we based on a procedure described previously [78]. This procedure was applied in all samples in which presence of classical or atypical PrP^res^ was uncertain: all TgShI112 animals developing disease spontaneously or inoculated both with atypical scrapie, ShTgSPON inoculum and sheepBSE; and also in TgVole, Tg338 and Tg501 animals inoculated either with atypical scrapie isolates or ShTgSPON inoculum. Briefly, 10% brain homogenates were digested with Pronase E (Sigma-Aldrich) at 100 µg/ml for 30 min at 37 °C and vigorous shaking (800 rpm). After addition of EDTA (Calbiochem) for a final concentration of 10 mM and Sarkosyl for a final 2% (w/V) concentration, Pronase E-digested samples were further processed with Benzonase (Merck) at 50 U/ml for 10 min at 37 °C and 800 rpm. Then, sodium phosphotungstic salt (NaPTA) (Sigma-Aldrich) was added at 0.3% (w/V) and samples incubated for 30 min at 37 °C and 800 rpm. Upon addition of iodixanol 60% (OptiPrep density gradient medium, Sigma-Aldrich) and NaPTA, for final concentrations of 35% (w/V) and 0.3% (w/V), respectively, samples were centrifuged at 16 100 g for 90 min and supernatant (avoiding flocculants) transferred to a new tube after filtration through 0.45 µm porus-size microcentrifuge filtration units (Millipore). These supernatants were afterwards mixed 1:1 with a buffer composed by 2% Sarkosyl (w/V) and 0.3% NaPTA diluted in PBS. After an additional 90 min centrifugation at 16 100 g the supernatant was discarded and pellet resuspended in washing buffer [iodixanol 17.5% (w/V) and Sarkosyl 0.1% (w/V) in PBS]. The resuspended pellets were then digested with Proteinase K at a final concentration of 10 µg/ml for 1 h at 37 °C and 800 rpm. After adding washing buffer and NaPTA for a final concentration of 0.3% (w/V), samples were once more centrifuged at for 30 min at 16 100 g and supernatants discarded. This step was repeated and the final pellet resuspended in loading buffer (NuPage 4X Loading Buffer, Invitrogen) 1:3 (V/V).

Prion protein immunodetection was performed by Western blotting as described previously [36]. Briefly, PK-digested samples were boiled for 10 min and loaded on 4–12% acrylamide gels (NuPAGE Midi gel Invitrogen Life Technologies) with the exception of the gel shown in Fig. 2, that was a 4–15% acrylamide gel (Criterion TGX, Bio-Rad), subjected to electrophoresis for approximately 1 h and 20 min and transferred to a PVDF membrane (Trans-Blot Turbo Transfer Pack, Bio-Rad) using the Trans-Blot® TurboTM transfer system (Bio-Rad). After blocking non-specific antibody binding of the membranes by incubation in 5% non-fat milk powder for 1 h at room temperature, monoclonal antibodies D18 (1:5000) [80] or 12B2 (1:5000) (Central Veterinary Institute, Wageningen UR) were added and incubated for 1 h at room temperature, prior to washing. After incubation with peroxidase-conjugated secondary goat anti-human IgG (H+L, Thermo Scientific or anti-mouse antibody (m-IgGκ BP-HRP, Santa Cruz Biotechnology)), membranes were washed again and developed with an enhanced chemiluminescent horseradish peroxidase substrate (West Pico Plus, Thermo Scientific), using a FluorChem Q (Alpha Innotech) for image acquisition and the software AlphaView (Alpha Innotech) for image processing.

For bank vole samples WB analysis, brain homogenates (20% w/v) were prepared as previously described [62]. After adding an equal volume of 100 mM Tris–HCl containing 4% sarkosyl, the homogenates were incubated for 30 min at 37 °C with gentle shaking. Proteinase K (Sigma-Aldrich) was added at a final concentration of 50 μg/ml and then the samples were incubated for 1 h at 55 °C with gentle shaking. Protease treatment was stopped with 3 mM PMSF (Sigma-Aldrich). Aliquots of samples were added with an equal volume of isopropanol/butanol (1:1 v/v) and centrifuged at 20 000 g for 5 min. Supernatants were discarded and the pellets were resuspended in denaturing sample buffer (NuPAGE LDS Sample Buffer, Invitrogen) and heated for 10 min at 90 °C. After electrophoresis on 12% bis–Tris polyacrylamide gels (Invitrogen) and WB on polyvinylidene fluoride membranes using the Trans-Blot Turbo Transfer System (Bio-Rad), the blots were processed with anti-PrP mAbs by using the SNAP i.d. 2.0 system (Millipore). Membranes were probed with mAbs SAF84 (aa 167–173, sheep numbering) or 9A2 (aa 102–104). The PrP was visualized by enhanced chemiluminescent substrate (SuperSignal Femto, Pierce) and the ChemiDoc imaging system (Bio-Rad).

### Ethics statement

All experiments involving mice (TgShI112, Tg340, Tg361, Tg338, and Tg501) were approved by the animal experimentation ethics committee of the Autonomous University of Barcelona (Reference numbers: 5767 and 1124M2R) in agreement with Article 28, sections a), b), c) and d) of the “Real Decreto 214/1997 de 30 de Julio” and the European Directive 86/609/CEE and the European Council Guidelines included in the European Convention for the Protection of Vertebrate Animals used for Experimental and Other Scientific Purposes.

TgVole (1x) and TgVole (4x) mice were obtained from the breeding colonies at CIC bioGUNE (Spain) and were inoculated at the University of Santiago de Compostela and Neiker—Basque Institute for Agricultural Research and Development. All experiments involving animals in Spain adhered to the guidelines included in the Spanish law “Real Decreto 1201/2005 de 10 de Octubre” on protection of animals used for experimentation and other scientific purposes, which is based on the European Directive 86/609/EEC on Laboratory Animal Protection. The project was approved by the Ethical Committees on Animal Welfare (project codes assigned by the Ethical Committee P-CBG-CBBA-0314 and 15,005/16/006) and performed under their supervision.

Bank voles were obtained from the breeding colony at the Istituto Superiore di Sanità (ISS), Italy. Experiments involving animals adhered to the guidelines contained in the Italian Legislative Decree 116/92, which is based on the European Directive 86/609/EEC on Laboratory Animal Protection, and then in the Legislative Decree 26/2014, which transposed the European Directive 2010/63/UE on Laboratory Animal Protection. The research protocols were approved and supervised by the Service for Biotechnology and Animal Welfare of the ISS and were authorized by the Italian Ministry of Health (decree numbers 84/12.B and 1119/2015-PR).

Experimental inoculation in sheep was approved by the Advisory Ethics Commission for Animal Experimentation of the University of Zaragoza with permit number PI 04/17. The care and use of animals was carried out in accordance with the Spanish Protection Policy Animal collected in RD 53/2013, which complies with the directive of the European Union 2010/63/EU regarding the protection of animals used for experimentation and other scientific purposes.

## Results

### Generation of a new transgenic mouse model with the ORF from sheep *PRNP* and the I112 polymorphism

Based on previous models set up in our laboratory, new mouse lines were generated by pronuclear injection of a construct consisting of the mouse PrP promoter and the Open Reading Frame (ORF) from sheep *PRNP* sequence with the I112 polymorphism, equivalent to polymorphism I109 in bank voles and identical to the *PRNP* from the Tibetan sheep breed. Five founders were obtained that transmitted the transgene to their progeny. After backcrossing to a line that did not express endogenous PrP (*Prnp*^−/−^ mice)[47], expression levels of the transgene were analyzed by *Western blotting* (WB) and two lines were selected: L456 and L460 with PrP^C^ expression levels of 1.5–2 × and 1–1.5 × in homozygosity, respectively, with an unaltered glycoform ratio upon WB (Additional file 1: Fig. S1).

### TgShI112 mice spontaneously develop a prion disease

**Fig. 1:**
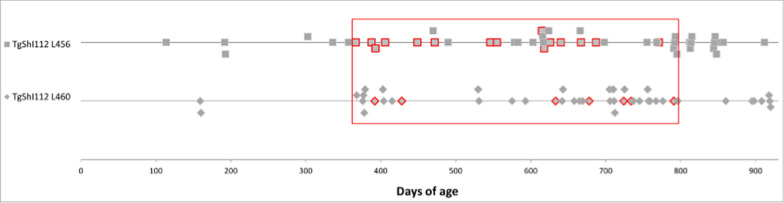
Graphical representation of the age of euthanasia of animals from TgShI112 L456 and L460 mouse lines. Every dot in the graph represents a mouse at the time it was euthanized. Dots with a red margin indicate TSE confirmed animals (by IDEXX ELISA, histopathology, and immunohistochemistry), all of which showed neurological clinical signs (except one animal that was found dead without previous signs, and two other animals that were euthanized due to a lung neoplasia and a massive bilateral hydrometra, respectively, and resulted positive in postmortem tests). A window (red rectangle) of time has been identified between 380 and 800 days of age (doa) in which clinical signs of a spontaneous TSE appear in both lines of the TgShI112 model. The remaining negative animals were euthanized for humanitarian reasons (i.e., final point criteria were reached due to signs unrelated to TSE) or in a programmed manner to obtain information at different time points

After an incubation period ranging from 380 to 800 days of age, 27% (15/52) of the animals of the L456 line and 15% (7/48) of the L460 line developed a prion disease, as confirmed by postmortem tests. All of them, except 3 that were euthanized due to intercurrent pathologies and were later confirmed as TSE positive, developed neurological signs characterized by a marked ataxia with titubation (when the animal attempted to walk a lateral swaying was observed that progressed to falling sideways in more advances cases), this lesion was consistent with a cerebellar location of the neurological lesion. Indeed moderate spongiosis was observed in the cerebellum with loss of neurons in the granular cell layer (Fig. 1).

**Fig. 2:**
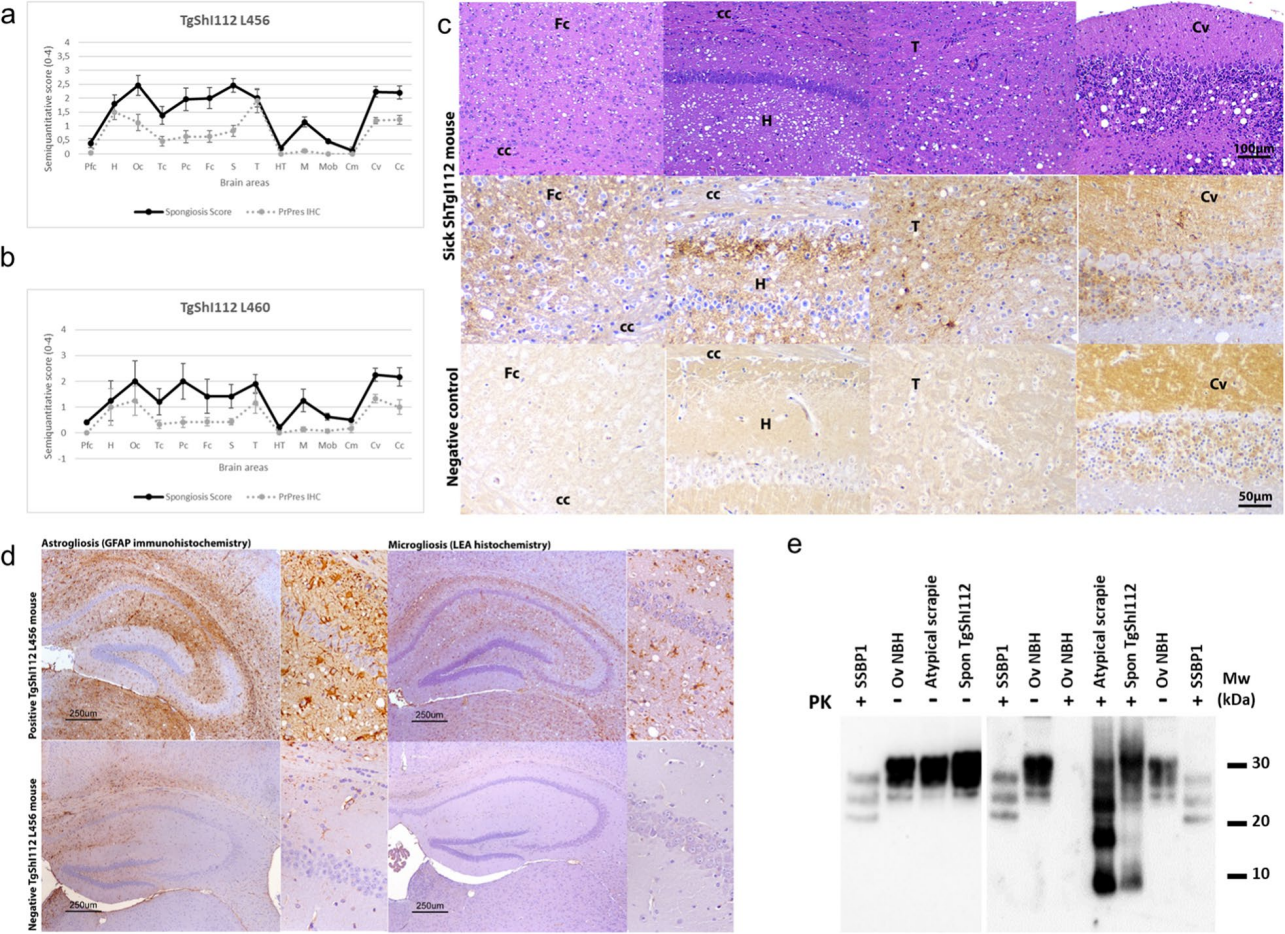
Brain lesions and PrP^res^ deposit distribution for TgShI112 mice. **a** Line 456 (n = 14) and **b** Line L460 (n = 7). Brain lesion profiles and PrP^res^ deposition profiles represent the mean semi-quantitative scoring (0–4, vertical axis) of the spongiform lesions (continuous line, black) and the immunohistochemical labelling of PrP^res^ deposits (dashed line, grey) against 14 brain regions (Pfc: piriform cortex, H: hippocampus, Oc: occipital cortex, Tc: temporal cortex, Pc: parietal cortex, Fc: frontal cortex, cc: corpus callosum; S: striatum, T: thalamus, HT: hypothalamus, M: mesencephalon, Mob: medulla oblongata, Cm: cerebellar nuclei, Cv: cerebellar vermis, Cc: cerebellar cortex). Lesions in line L460 are milder than those of L456 but follow a similar distribution. Bars: standard error of the mean. **c** Histopathological assessment of spongiform lesions and PrP^res^ deposits in TgShI112 mice (line 456).Spongiform change can be observed in the top row (H&E staining). PrP^res^ deposits are of punctiform and granular nature. In the bottom row a healthy L456 mouse brain is shown. Notice the intense PrP^C^ backroad in the cerebellar molecular layer and the synaptic glomerules (but lacking the punctiform PrP^res^ staining pattern). Mouse monoclonal antibody 2G11 (1:100). **d** Neuroinflammatory response. The animals that develop a disease also show a conspicuous neuroinflammatory response with activation of both astroglial (Glial fibrillary acidic protein –GFAP- immunohistochemistry, 1:1500) and microglial (*Lycopersicum esculentum* agglutinin –LEA- affinity histochemistry, 1:50) cell populations in comparison to age matched unaffected siblings. **e** Western blotting of a classical scrapie isolate (SSBP/1) and a healthy sheep brain homogenate (Ov NBH) digested for the detection of classical PrP^res^, compared to an isolate of atypical (Nor98) ovine scrapie and the prions extracted from spontaneously diseased TgShI112 mice digested according to the protocol described in [78], which allows detection of atypical PrP^Sc^ pattern, and requires 20 times more brain homogenate. The same undigested samples from spontaneously diseased TgShI112 mice and atypical scrapie were also included. Anti PrP antibody 12B2 (1:5000). Spontaneous TgShI112 displays an atypical ladder-like pattern resembling the Nor98 atypical scrapie isolate. MW: Molecular weight

Upon necropsy, the brains of the animals were harvested and a positive result to enzyme immunosorbent assay for the detection of PrP^res^ (IDEXX herdcheck) was obtained in all animals with clinical signs. Neuropathological examination revealed lesions compatible with a prion disease, namely spongiosis, astro- and microgliosis, and deposition of PrP^res^ aggregates (Fig. 2).

Spongiform change was prominent in the cerebellar cortex, mesencephalon colliculi, thalamus, hippocampal formation, neocortex (frontal, parietal, and occipital lobes) and striatum. The medulla oblongata, mesencephalon tegmentum, hypothalamus, and piriform cortex were mostly spared. Fine punctate PrP^res^ immunolabelling was observed in the cerebellar cortex with seldom coarse granular aggregates, in the thalamus, particularly intense in the dorsolateral nuclei, and in the hippocampal formation, selectively involving the *alveus* and *lacunosum moleculare* layers. A certain degree of variation was observed in the intensity of the lesions and PrP^res^ immunostaining. Some animals (mostly those of the L456 line) showed widespread involvement of the neocortical lobes whether in other animals a milder phenotype was observed confined to the cerebellar cortex and mildly involving the thalamus and hippocampal formation. No correlation was found between the age at which signs appeared and this variability, since some of the animals with widespread lesions were younger than those with the milder phenotype (Spearman r = 0.1690, P = 0,6216). The lesion type and distribution were very similar in both lines but lesions in animals from line L460 were milder, meaning that among the positive animals of this line, a lesser proportion of animals with widespread lesions was found (40% vs. 28%) (Fig. 2). Interestingly, a mild punctate PrP^res^ staining was observed in the white matter.

The biochemical analysis by WB of the prion protein was performed after digestion with proteinase K of the brain homogenates from mice with clinical signs showing an atypical ladder-like pattern resembling the one observed in Nor98 atypical scrapie isolates, characterized by a low molecular weight fragment of 7–10 kDa (Fig. 2e).

### The prions generated in the TgShI112 model are transmissible

To assess whether the phenotype observed was a *bona fide* prion disease, we needed to demonstrate that the new spontaneous prions were transmissible. With this goal in mind, an inoculum (termed ShTgSPON) was prepared from a pool of brain homogenates of five positive animals form the L456 line, which was intracerebrally inoculated in both L460 and L456 lines of the same TgShI112 model. All inoculated animals succumbed to a prion disease with an incubation period much shorter than the minimum 380 days of age required for the spontaneous phenotype to appear: 162 ± 12 days post inoculation (dpi) (standard error of the mean -s.e.m.-); for the L460 line and a little shorter, 152 ± 11 dpi ± s.e.m., for the L456 line (Table 1).

**Table 1:** Summary of the bioassays performed in TgShI112 mice. Mean dpi of positive animals ± SEM (positive/inoculated). NP: Not performed

	TgShI112 L460	TgShI112 L456
	1st passage	1st passage
ShTgSPON	162 ± 12 (8/8)	152 ± 11 (9/9)
SSBP/1	> 570 (0/10)^a^	572 (1/5)^b^
Atypical Scrapie	ND	177 ± 14 (14/14)
Sheep BSE	590 ± 20 (6/6^c^)	ND

^a^One animal was euthanized at 380 dpi with spontaneous atypical profile. Endpoint established at 579 dpi

^b^In one animal a fine punctate PrP^res^ signalling was observed in the medulla oblongata, absence of atypical profile. IDEXX Positive

^c^A seventh animal was prematurely euthanized at 352 dpi and was negative, it was excluded from the calculations. ND: not determined

**Fig. 3:**
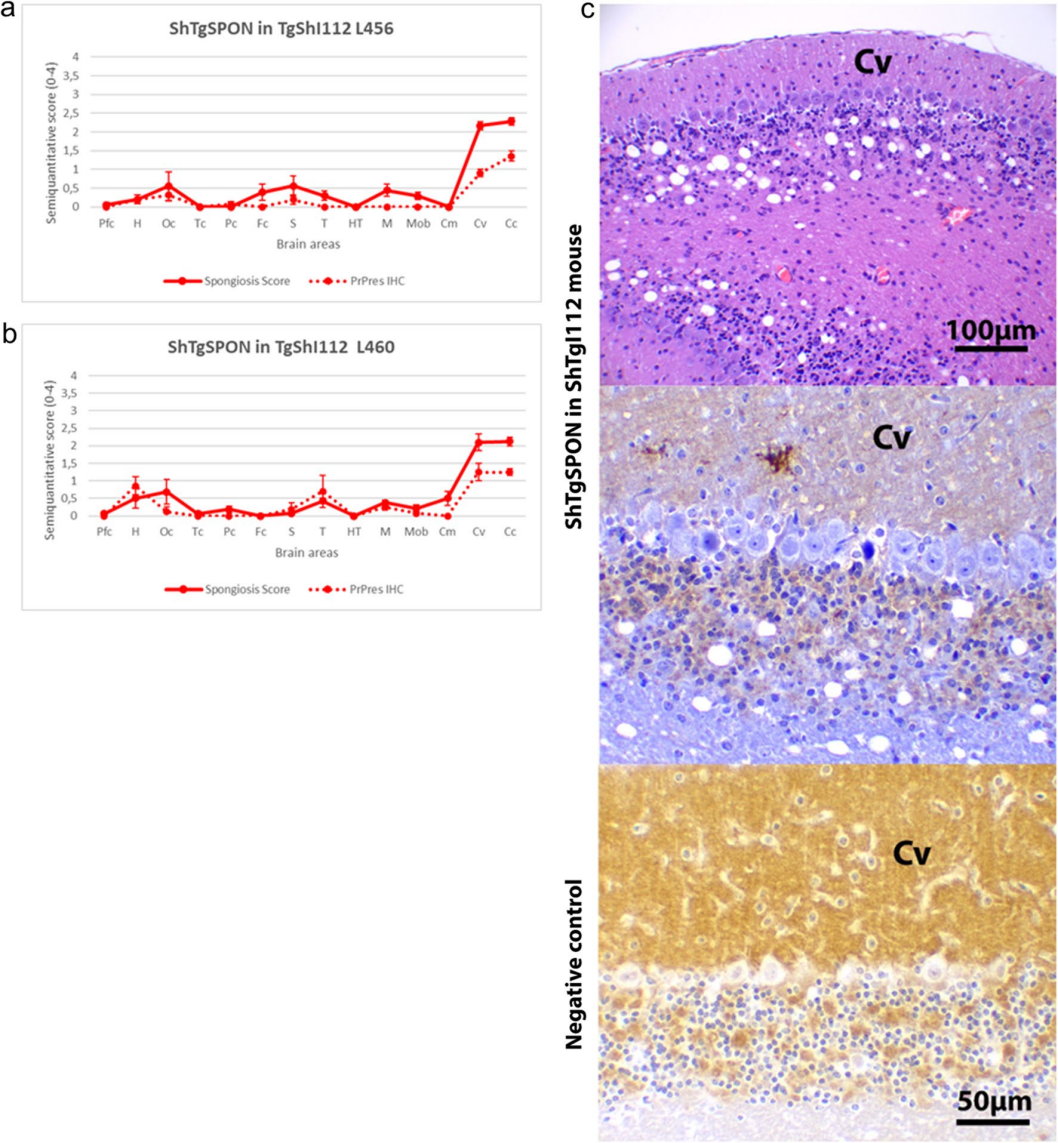
Brain lesion profiles and PrP^res^ deposit distribution for the inoculum ShTgSPON in TgShI112 mice. **a** Line 456 (n = 9) and **b** line L460 (n = 8). Brain lesion and PrP^res^ deposition profiles represent the mean semi-quantitative scoring (0–4, vertical axis) of the spongiform lesions (continuous line, red) and the immunohistochemical labelling of PrP^res^ deposits (dashed line, red) against 14 brain regions (*Pfc* Piriform cortex, *H* Hippocampus, *Oc* Occipital cortex, *Tc* Temporal cortex, *Pc* Parietal cortex, *Fc* Frontal cortex, *S* Striatum, *T* Thalamus, *HT* Hypothalamus, *M* Mesencephalon, *Mob* Medulla oblongata, *Cm* Cerebellar nuclei, *Cv* Cerebellar vermis, *Cc* Cerebellar cortex). Lesions and PrP^res^ deposition are mostly restricted to cerebellar cortex and vermis, a profile clearly distinguishable from the spontaneous phenotype. Bars: standard error of the mean, **c** Neuropathological characterisation of the lesions (H&E staining) and PrP^res^ immunohistochemistry (2G11, 1:100) in the brains of ShTgSPON inoculated TgShI112 (L456) mice. Spongiosis and evident neuronal loss was observed in the granular cell layer of the cerebellar cortex (the cerebellar vermis -Cv- is depicted)) along with punctate-granular PrP^res^ deposit in the cerebellar cortex and vermis. A healthy L456 brain mouse is shown as a negative control, notice a strong PrP^C^ background in the molecular layer and the synaptic glomerules and lack of granular PrP^res^ staining pattern

The lesions and PrP^res^ distribution were similar to the milder phenotype but differed from the widespread pattern observed in the spontaneous phenotype, being mostly restricted to the cerebellar cortex and vermis with an evident loss of granules from the granular layer (Fig. 3). The immunostaining pattern observed also consisted of fine granular to small clusters of coarse granular PrP^res^ deposits.

### ShTgSPON is indistinguishable from atypical scrapie when bioassayed in TgShI112 mice

**Fig. 4:**
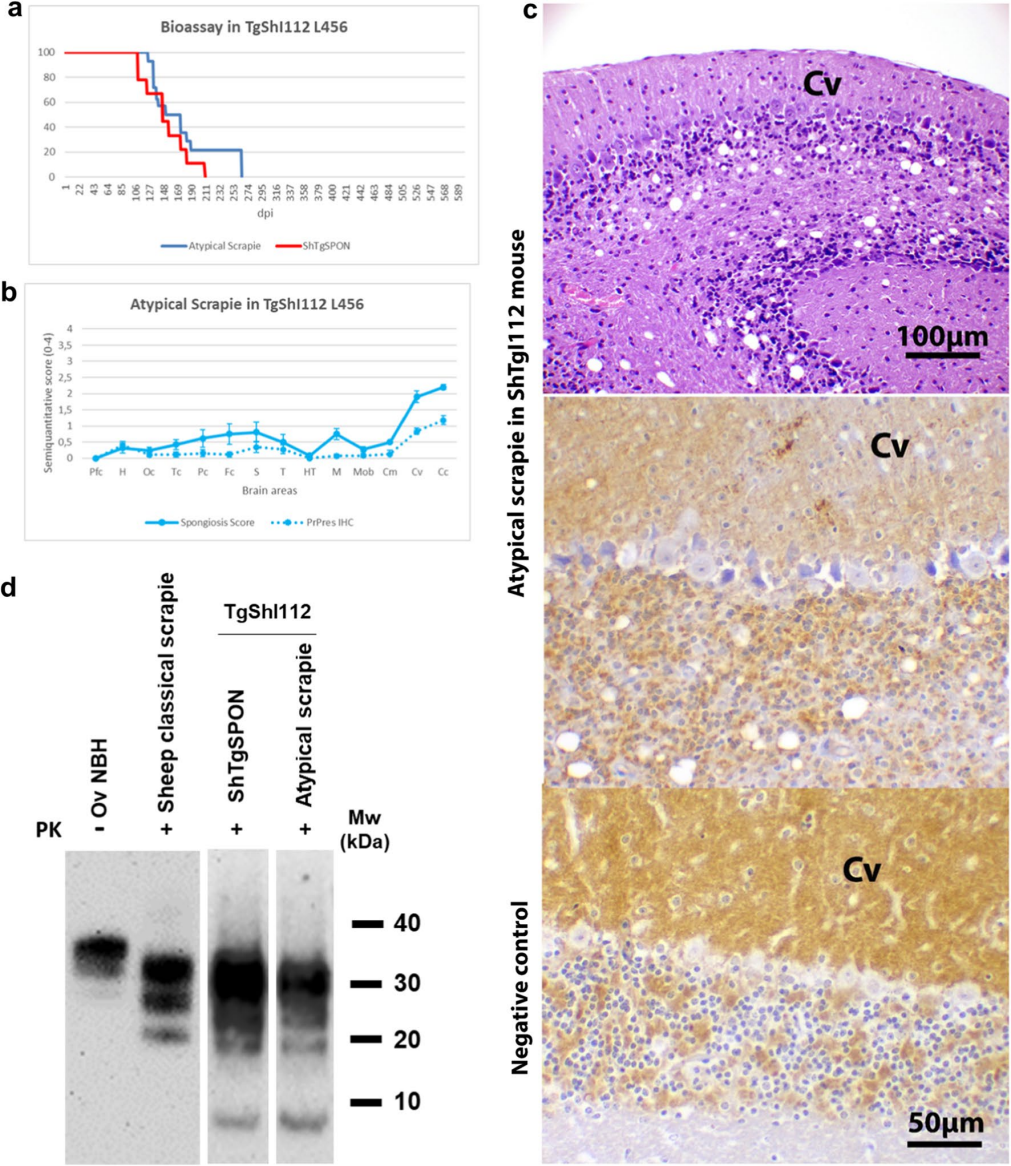
Analysis of ShTgSPON-inoculated compared to atypical scrapie-inoculated TgShI112 mice. **a** Kaplan-Meyer survival curves comparing ShTgSPON (red) innoculum and an atypical scrapie isolate (blue) incubation periods in the TgShI112 mouse model. Notice the overlapping curve. *dpi* Days post inoculation. **b** Brain lesion and PrP^res^ deposit distribution for the atypical scrapie inoculum in TgShI112 mice (line 456, n = 14). Brain lesion profiles and PrP^res^ deposition profiles represent the mean semi-quantitative scoring (0–4, vertical axis) of the spongiform lesions (continuous line, blue) and the immunohistochemical labelling of PrP^res^ deposits (dashed line, blue) against 14 brain regions (*Pfc* Piriform cortex, *H* Hippocampus, *Oc* Occipital cortex, *Tc* Temporal cortex, *Pc* Parietal cortex, *Fc* Frontal cortex, *S* Striatum, *T* Thalamus, *HT* Hypothalamus, *M* Mesencephalon, *Mob* Medulla oblongata, *Cm* Cerebellar nuclei, *Cv* Cerebellar vermis, *Cc* Cerebellar cortex). Lesions and PrP^res^ deposits were mostly restricted to the cerebellar cortex and vermis. This pattern was identical to that observed in the same mice when inoculated with ShTgSPON inoculum (see Fig. 3a and b). Bars: standard error of the mean. **c** Neuropathological characterisation of the lesions (H&E staining) and PrP^res^ immunohistochemistry (2G11, 1:100) in the brains of atypical scrapie inoculated TgShI112 mice (L456). Spongiosis and evident neuronal loss was observed in the granular cell layer of the cerebellar cortex (the cerebellar vermis is depicted -Cv-) along with punctate-granular PrP^res^ deposit in the cerebellar cortex and vermis. This pattern was identical to that observed in the same mice when inoculated with ShTgSPON inoculum (see Fig. 3c). A healthy L456 brain mouse is shown as a negative control, notice a strong PrP^C^ background in the molecular layer and the synaptic glomerules and lack of granular PrP^res^ staining pattern. (d) Western blotting comparison of a healthy sheep brain homogenate (Ov NBH), a classical scrapie isolate (SSBP/1), digested for detection of classical PrP^res^, and the prions extracted from spontaneously diseased TgShI112 mice or atypical scrapie inoculated TgShI112 mice after digestion with proteinase K (PK) following the protocol described in [78]. Anti PrP antibody 12B2 (1:5000). Electrophoretic migration profiles of both, the spontaneously developed prions in TgShI112 mice (ShTgSPON prions) and an ovine atypical scrapie isolate inoculated in TgShI112 animals, are indistinguishable and are characterized by a low molecular weight band of around 7–10 kDa, further indicating the similarity of the spontaneously generated prions with atypical scrapie isolates. The presence of higher molecular weight bands in these samples, apart from the characteristic 7–10 kDa fragment, could be due to the presence of mildly protease resistant forms that arise due to the low PK concentration and the requirement of using large amounts of brain homogenate for the detection of the small fragment. MW: Molecular weight

Since the biochemical signature of the PrP^res^ from spontaneously sick animals was remarkably similar to that of natural isolates from sheep with atypical scrapie, an isolate of ovine atypical scrapie (a field case diagnosed in PRIOCAT laboratory, IRTA-CReSA, Catalonia) was inoculated in the same model TgShI112 (line 456) to compare it with the spontaneous disease. This bioassay resulted in a 100% attack rate at 177 ± 14 dpi ± s.e.m. (Table 1, Fig. 4a), an incubation period similar to that observed for ShTgSPON (100% attack rate at 152 ± 11 dpi ± s.e.m.). The brain lesion and PrP^res^ distribution profiles were indistinguishable from those obtained in the same model when inoculated with the ShTgSPON inoculum (Fig. 3. Again, the biochemical profile on WB matched the multi-band pattern observed in atypical scrapie (Fig. 4d).

### Propagation of classical prion diseases is not precluded by I112 polymorphism in ovine PrP^C^

To assess whether the I112 polymorphism could restrict prion propagation only to those with atypical features or, on the contrary, could be also permissive to classical prion disease propagation and modelling, TgShI112 mice from lines L456 (n = 5) and L460 (n = 10) were intracerebrally inoculated with SSBP/1, a pool of brain homogenates from sheep affected by classical scrapie and also with a sheepBSE inoculum. SSBP/1 inoculated animals were culled at 570 dpi without any clinical signs. No pathological or biochemical indication of a prion disease was observed except for one animal (line 456), in which a focal area of punctate PrP^res^ immunolabelling was observed in the medulla oblongata. This animal had an ELISA positive result in absence of the spontaneous phenotype described previously. This result is in accordance with other ovine models with the ARQ genotype that show reduced susceptibility to the SSBP/1 classical scrapie isolate [34, 40]. In contrast, the six TgShI112 (Line 460) animals inoculated with a sheep-BSE inoculum succumbed to disease with a 100% attack rate and 590 ± 20 dpi ± s.e.m.. Even though this prolonged incubation period could overlap with the spontaneous phenotype in some animals, the immunostaining pattern, consisting of multifocal round plaque-like PrP^res^ deposits, the distribution of lesions in the brain confirmed that a classical prion strain was being replicated in the model (Table 1, Additional file 1: Fig. S2). The length of this incubation period is comparable to that reported in other ARQ ovine models such as Tg501 (485 ± 62 dpi ± s.e.m.)[3]. Since the Tg501 model slightly overexpresses ovine ARQ PrP^C^ it is reasonable that the incubation period is slightly shorter than in our model.

### Transmission of ShTgSPON into mouse models expressing wild type ovine PrP demonstrates it is indistinguishable from atypical scrapie

#### ***ShTgSPON inoculation in the ovine VRQ PrP***^***C***^*** Tg338 model***

The transgenic mouse model Tg338 [8, 69] bearing the wild type ovine *PRNP* gene with VRQ polymorphisms, which easily propagates Nor98 isolates [24], was inoculated with both ShTgSPON and the atypical scrapie isolate (Table 2). On first passage, a 100% attack rate demonstrated that ShTgSPON prions were also transmissible to a wild type ovinized mouse model.

**Table 2:** Summary of the bioassays performed in mice to compare the ShTgSPON inoculum with atypical scrapie and SSPB/1. Mean dpi of positive animals ± SEM (positive/inoculated). NP: Not performed

	Tg338 (ovine VRQ PrP^C^)		Tg501 (ovine ARQ PrP^C^)	TgVole 1x	TgVole 4x	Bank vole
	1st passage	2nd passage	1st passage	1st passage	1st passage	1st passage
ShTgSPON	172 ± 5 (11/11)	177 ± 8 (10/10)	455 ± 14 (12/12)	120 ± 1 (13/13)	59 ± 2 (5/5)	256 ± 14 (12/12)
SSBP/1	72 ± 1 (11/11)	ND	502 ± 27 (5/5)	ND	ND	523 ± 62 (9/10)
Atypical scrapie	239 ± 2 (12/12)	144 ± 3 [41]^b^	577 ± 7 (5/5)^a^	135 ± 6 (17/17)	80 ± 2 (4/4)	247 ± 24 (9/9)^c^

^a^A sixth animal was found death at 104 dpi and was excluded from the study

^b^Using 10 times more inoculum (10% vs. 1%) than in our inoculations. *ND* Not determined

^c^Data published by Pirisinu et al. [61]

**Fig. 5:**
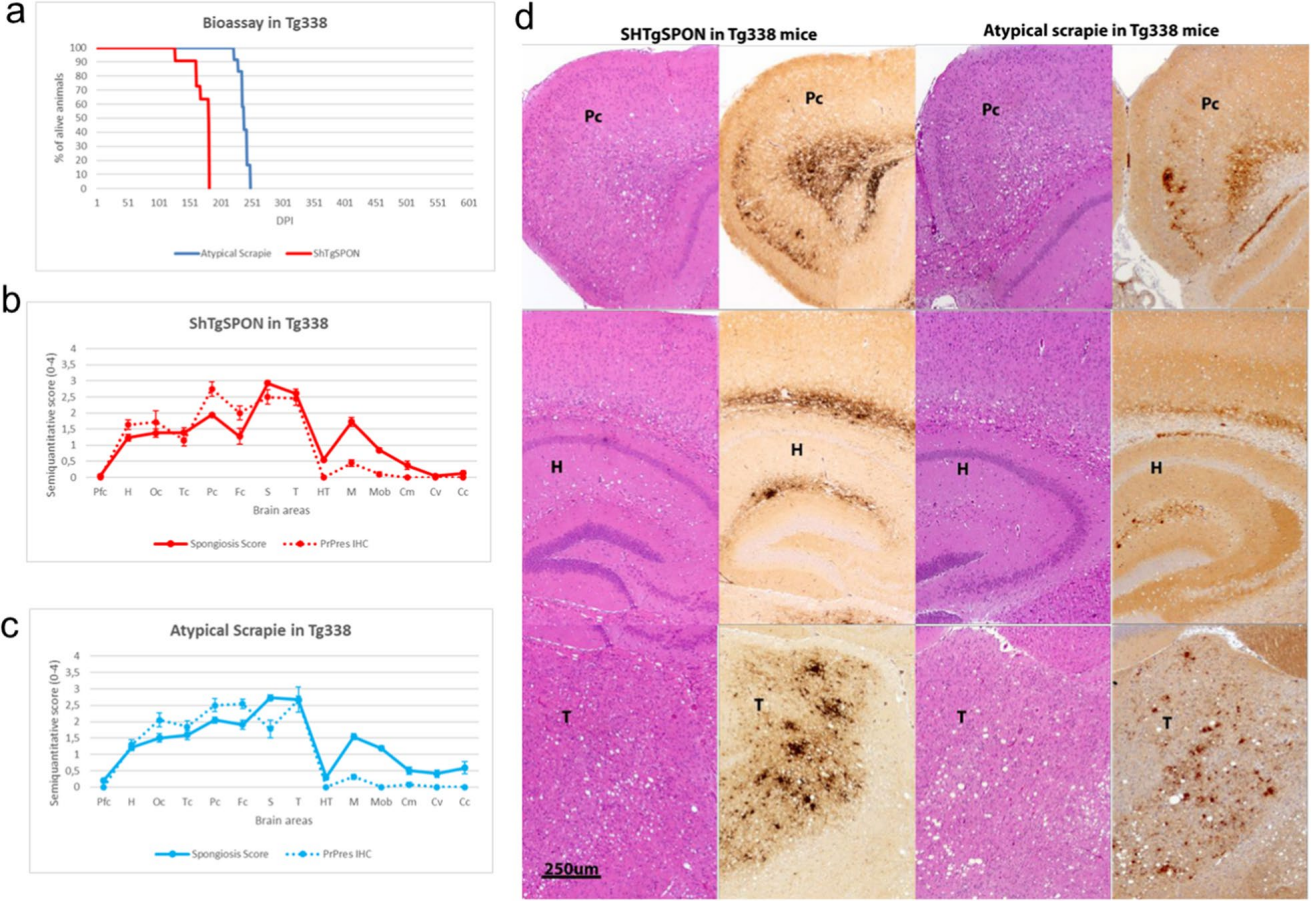
Anatomopathological analysis of ShTgSPON-inoculated compared to atypical scrapie-inoculated Tg338 mice (Ovine VRQ PrP^C^). **a** Kaplan-Meyer survival curves comparing ShTgSPON (red) innoculum and an atypical scrapie isolate (blue) incubation periods in the Tg338 mouse model. **b** Brain lesion and PrP^res^ deposit distribution for the inoculum ShTgSPON (n = 11) and **c** atypical scrapie in Tg338 mice (n = 12). Brain lesion profiles and PrP^res^ deposition profiles represent the mean semi-quantitative scoring (0–4, vertical axis) of the spongiform lesions (continuous line, blue) and the immunohistochemical labelling of PrP^res^ deposits (dashed line, blue) against 14 brain regions (*Pfc* piriform cortex, *H* Hippocampus, *Oc* Occipital cortex, *Tc* Temporal cortex, *Pc* Parietal cortex, *Fc* Frontal cortex, *S* Striatum, *T* Thalamus, *HT* Hypothalamus, *M* Mesencephalon, *Mob* Medulla oblongata, *Cm* Cerebellar nuclei, *Cv* Cerebellar vermis, *Cc* Cerebellar cortex). Notice the striking similarity of the brain profiles obtained with both isolates. Bars: standard error of the mean. **d** Neuropathological characterisation of the lesions (H&E staining) and PrP^res^ immunohistochemistry (2G11, 1:100) in the brains of ShTgSPON (left panel) and atypical Scrapie (right panel) inoculated Tg338 mice. Both inocula show an identical lesional and PrP^res^ deposition pattern. Notice the selective involvement of both isolates of the different layers of the parietal cortex (*III-IV and VI*) and hippocampal formation (*alveus and lacunosum moleculare*) as well as that of the dorso-lateral thalamic nuclei

The incubation period for the ShTgSPON isolate was slightly shorter than for the atypical scrapie isolate, both with a 100% attack rate, observing a striking similarity regarding the spongiosis and PrP^res^ deposition brain profiles (Fig. 5) suggesting that both inocula contained the same prion strain. When compared to a classical scrapie strain, SSBP/1, the differences were substantial (Additional file 1: Fig. S3). Furthermore, on second passage of the ShTgSPON inoculum, no change was observed on the incubation period indicating a lack of transmission barrier or adaptation phenomena (Additional file 1: Fig. S3).

**Fig. 6:**
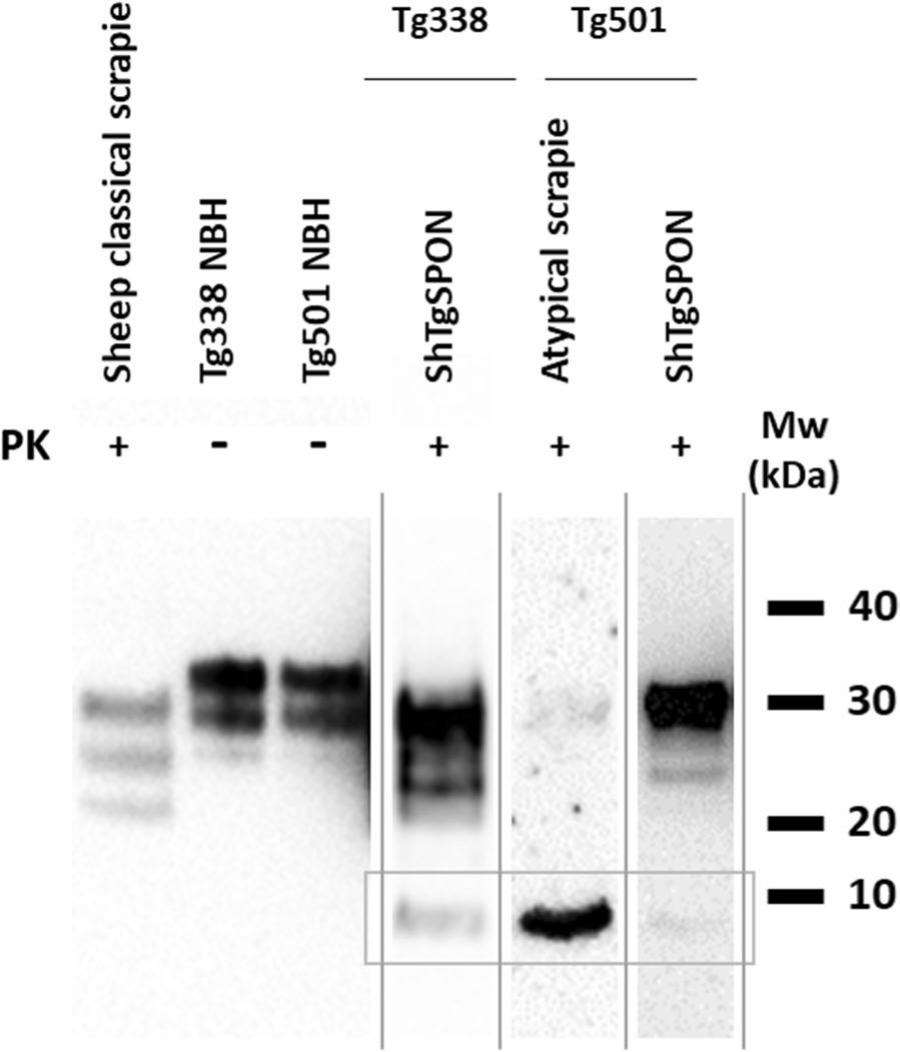
Western blotting comparison of a classical scrapie isolate (SSBP/1) and the prions extracted from diseased TgShI112 or atypical scrapie inoculated Tg338 (Ovine VRQ PrP^C^) and Tg501 (Ovine ARQ PrP^C^) mouse brains after digestion with proteinase K (PK). Electrophoretic migration profiles of prions from Tg338 and Tg501 mouse lines inoculated intracerebrally with ShTgSPON are similar and characterized by the presence of a 7–10 kDa band, also similar to the ovine atypical scrapie isolate inoculated in the same models. The detection of mildly PK-resistant high molecular weight bands in some of the samples may respond to some degree of variability due to the differences between the models in terms of PrP sequence, expression levels and slight variations in disease stage at culling. Along this line, variability due to the processing of the samples for the detection of the low molecular weight band could also explain the occasional presence of high molecular weight PrP fragments, given the low PK concentration and high sample amounts required. Since anatomopathological analysis did not show significant differences on plaque type and distribution between animals with or without them. Overall, all models challenged with ShTgSPON show the characteristic 7–10 kDa fragment resembling that from atypical scrapie, indicating the spontaneous formation of an atypical ovine prion in TgSh112I model. Anti PrP antibody 12B2, 1:5000. MW: Molecular weight

Biochemical analysis of the PrP^res^ from ShTgSPON inoculated animals further confirmed the transmission of an atypical prion disease in this model, characterized by the expected low molecular weight band of around 7–10 kDa (Fig. 6).

#### ***ShTgSPON inoculation in the ovine ARQ PrP***^***C***^*** Tg501 model***

**Fig. 7:**
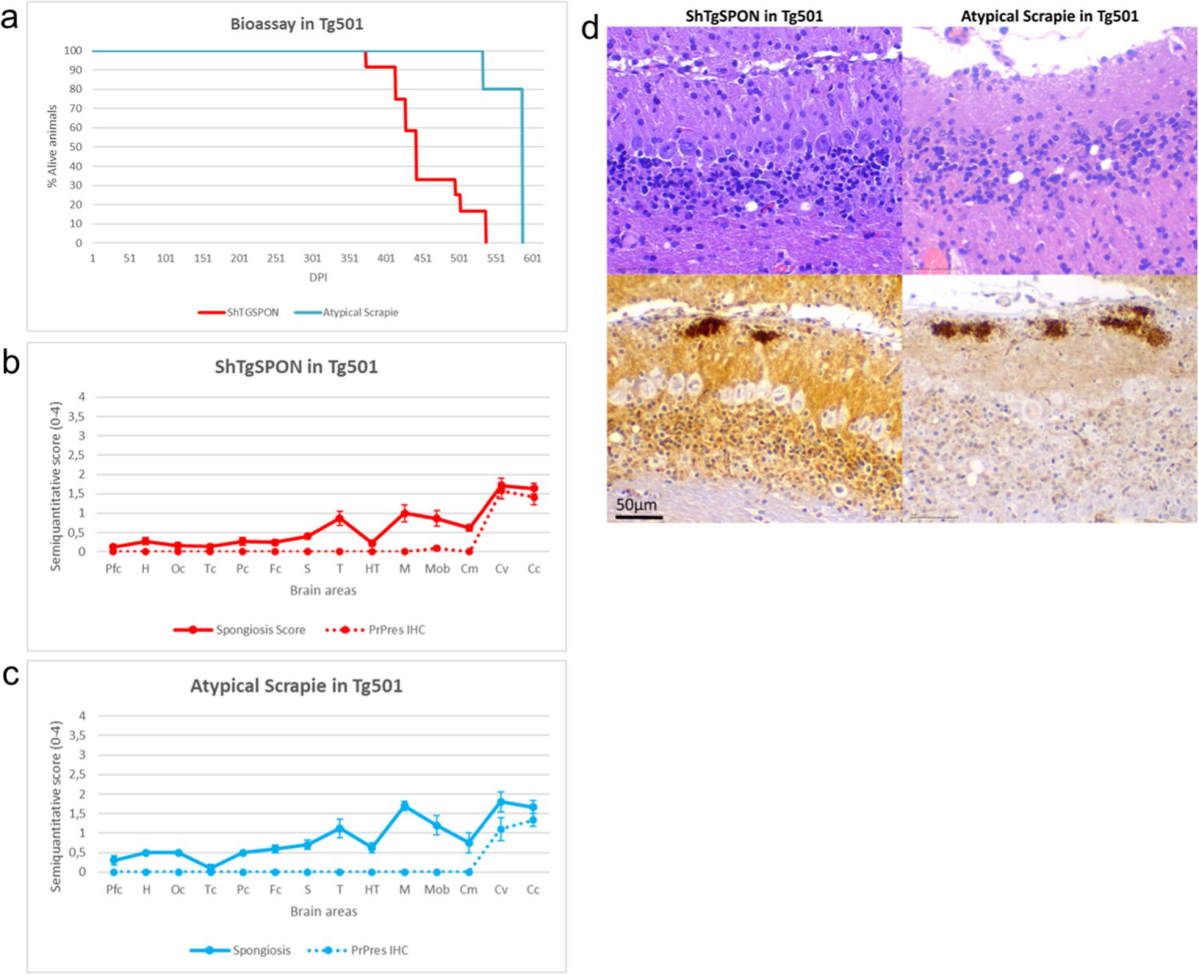
Anatomopathological analysis of ShTgSPON-inoculated compared to atypical scrapie-inoculated Tg501 mice. **a** Kaplan-Meyer survival curves comparing ShTgSPON (red) innoculum and an atypical scrapie isolate (TOA3, blue) incubation periods in the Tg501 mouse model. **b** Brain lesion and PrP^res^ deposit distribution for the inoculum ShTgSPON (n = 12) and **c** atypical scrapie (n = 5) in Tg501 mice. Brain lesion profiles and PrP^res^ deposition profiles represent the mean semi-quantitative scoring (0–4, vertical axis) of the spongiform lesions (continuous line, blue) and the immunohistochemical labelling of PrP^res^ deposits (dashed line, blue) against 14 brain regions (*Pfc* Piriform cortex, *H* Hippocampus, *Oc* Occipital cortex, *Tc* Temporal cortex, *Pc* Parietal cortex, *Fc* Frontal cortex, *S* Striatum, *T* Thalamus, *HT* Hypothalamus, *M* Mesencephalon, *Mob* Medulla oblongata, *Cm* Cerebellar nuclei, *Cv* Cerebellar vermis, *Cc* Cerebellar cortex). Notice the striking similarity of the brain profiles obtained with both isolates. Bars: standard error of the mean. **d** Neuropathological characterisation of the lesions (H&E staining) and PrP^res^ immunohistochemistry (2G11, 1:100) in the brains of ShTgSPON (left panel) and atypical scrapie (right panel) inoculated Tg501 mice. Both inocula show an identical lesional and PrP^res^ deposition pattern. Notice the selective involvement of both isolates of the cerebellar cortex with focal massive granule cell loss and plaque-like PrP^res^ deposits in the superficial molecular layer of the cerebellar cortex and vermis

A second ovinized transgenic mouse model was used bearing the wild type ovine *PRNP* gene with ARQ polymorphisms, the Tg501 [2–4]. As with the previous model, a 100% attack rate was obtained after inoculation with ShTgSPON and a slightly shorter incubation period than that of atypical scrapie isolate (Table 2). The brain spongiosis and PrP^res^ profiles were once again indistinguishable; spongiosis was minimal in both groups with a severe loss of granular neurons in the cerebellar granular cell layer, and the presence of PrP^res^ plaque-like deposits only in the cerebellar cortex and vermis (Fig. 7). These features contrast with the distribution of PrP^res^ and lesion profile of a classical scrapie strain, such as SSBP/1, in the same model, in which the cerebellar cortex is mostly spared, and lesions are centred in the brain stem (Additional file 1: Fig. S3).

Biochemically, the PrP^res^ pattern was also comparable to that obtained after inoculation with an atypical scrapie isolate in the same model, showing the characteristic low molecular weight band that is absent in classical scrapie isolates. Differences in the intensity of such fragment and the presence of higher molecular weight bands, reminiscent of the electrophoretic migration profile of classical scrapie isolates, likely derive from the processing of the samples for the detection of the low molecular weight band, which requires large amounts of brain homogenate, low PK concentration and exhaustive purification and concentration steps (Fig. 6).

### Inoculation of ShTgSPON and atypical scrapie in bank vole-based models reveals the facilitating role of isoleucine

Despite the differences in PrP^C^ expression levels of the three ovine models inoculated with atypical scrapie and ShTgSPON, the disease was unexpectedly fast in TgShI112 (with the lowest PrP^C^ expression levels among the three models used) pointing towards a relevant role of the I112 polymorphism. Since the particularity of the bank vole model relies on the presence of isoleucine at the equivalent position (position 109), which promotes the spontaneous formation of an atypical prion strain when overexpressed in transgenic mice and allows efficient transmission of atypical scrapie in bank voles [62, 75], we wondered whether the presence of this polymorphism could facilitate propagation of atypical prions. For that, we inoculated ShTgSPON and an atypical scrapie isolate in different animal models bearing bank vole PrP with I109 polymorphism (TgVole 1x, TgVole 4x, and bank voles) (Table 2). As reported in previous studies, bank voles with M109 polymorphism and wild type mice do not succumb to disease upon intracerebral inoculation of atypical scrapie or do so in a highly inefficient manner [35, 61]. Whereas the bank vole with I109 and our TgVole models, with 100% attack rate, manifested neurological signs in less than 260 dpi in non-overexpressing ones and around 100 dpi in those overexpressing PrP^C^ fourfold. Moreover, the incubation periods of ShTgSPON were similar to those of atypical scrapie inocula in the three models (Table 2).

**Fig. 8:**
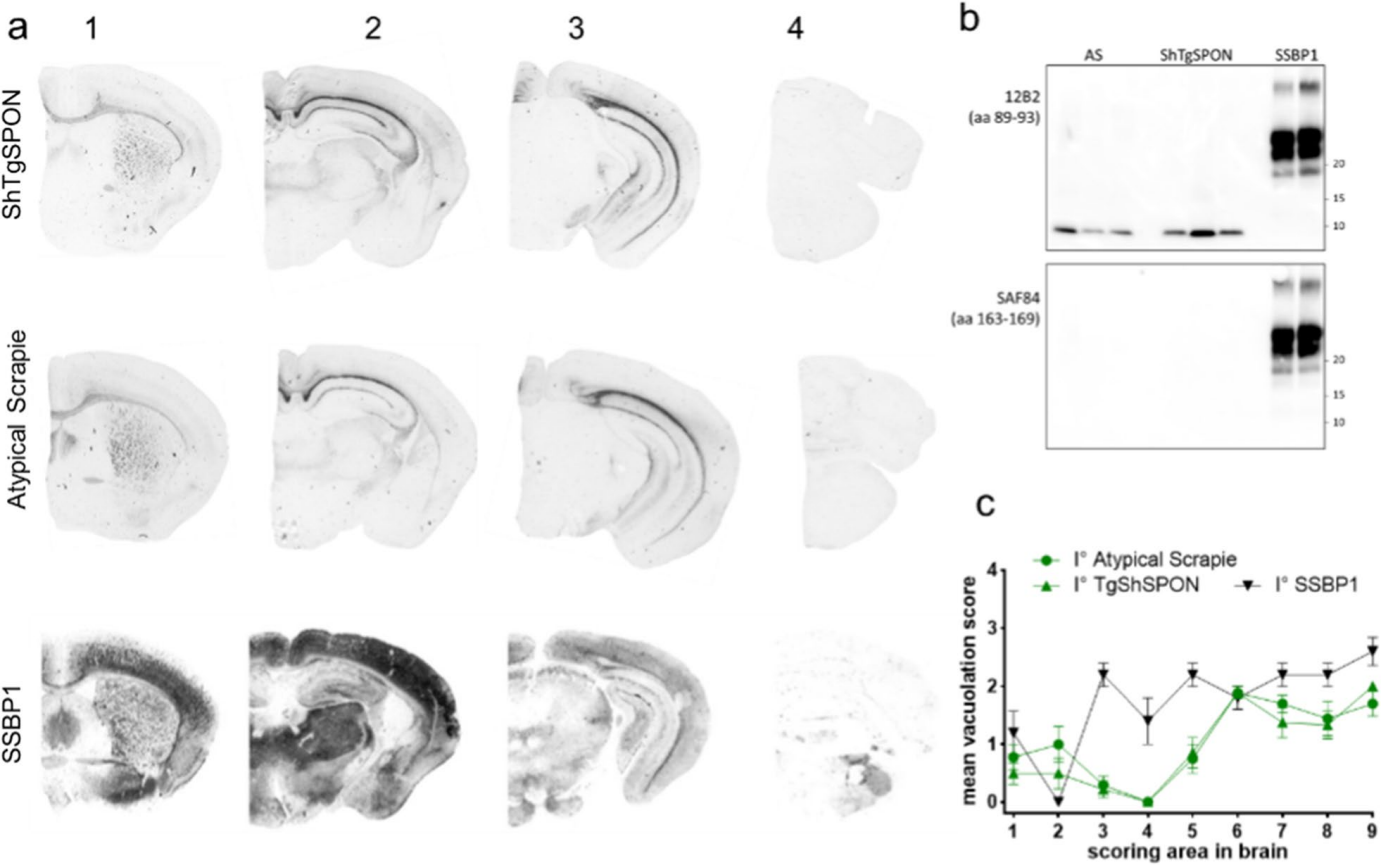
Bank vole bioassay. **a** PET blot detection of protease resistant PrP^Sc^ in coronal sections of the brain, representing telencephalon (1), diencephalon (2), midbrain (3), and hindbrain (4) from representative Bv109I. Isolates indicated on the *Left*. PrP^Sc^ was detected with mAb 6C2. **b** Representative replica Western blots showing PK-treated (PK) PrP^Sc^ from the brains of voles inoculated with atypical scrapie (AS, n = 9), ShTgSPON (n = 12) and classical scrapie (SSBP/1, n = 9), as indicated on the *Top* of the blots, and analyzed with mAbs recognizing different epitopes on PrP, as indicated on the *Left* of the blots (SAF84 epitope at 167 to 173; 12B2 epitope at 89 to 93). Notice absence of labelling for the c-terminal epitope for both AS and ShTgSPON. **c** Lesion profiles in groups of Bv109I infected with atypical scrapie (AS), ShTgSPON and classical scrapie (SSBP/1). Data points represent the mean ± SEM of at least five voles per group. Brain-scoring areas: medulla (1), cerebellum (2), superior colliculus (3), hypothalamus (4), thalamus (5), hippocampus (6), septum (7), retrosplenial and adjacent motor cortex (8), and cingulate and adjacent motor cortex (9)

**Fig. 9:**
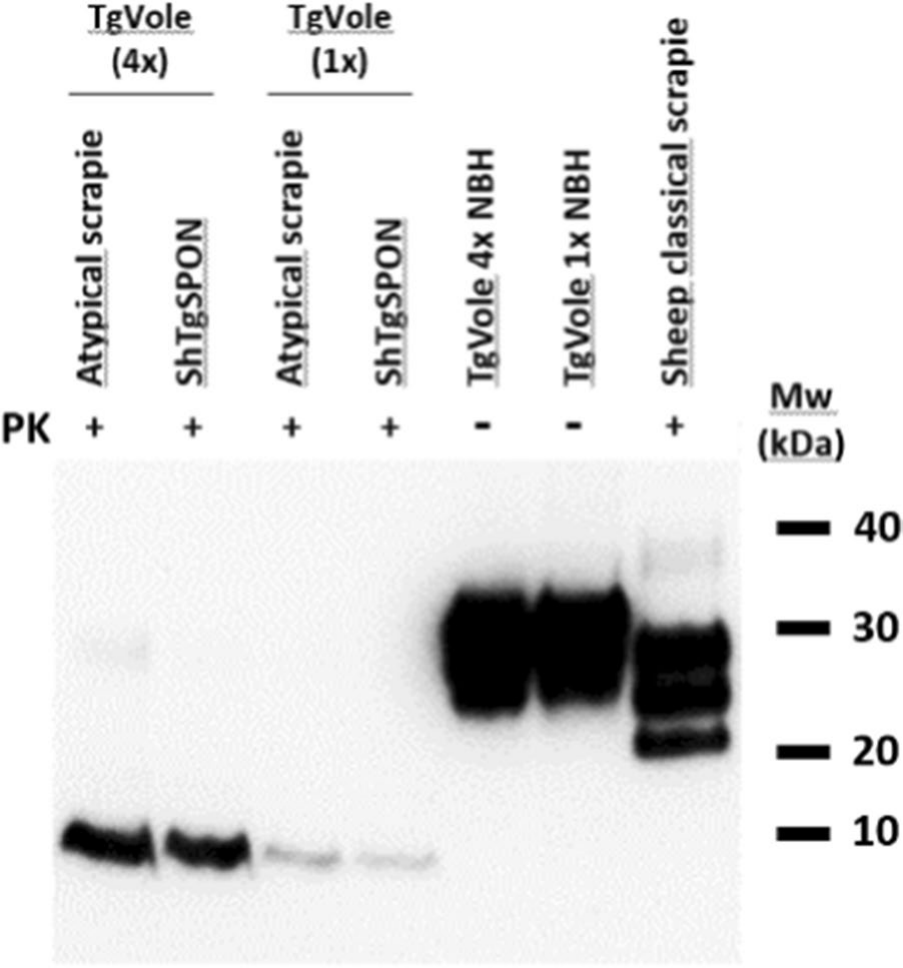
Western blotting comparison of a classical scrapie isolate (SSBP/1) and the prions extracted from spontaneous-diseased TgShI112 or atypical scrapie inoculated TgVole (4x) and TgVole (1x) mouse brains after digestion with proteinase K (PK). Electrophoretic migration profiles of prions from TgVole (1 × and 4x, with respect to mouse PrP expression levels) inoculated intracerebrally with ShTgSPON are similar and characterized by the presence of a 7–10 kDa band, also indistinguishable from ovine atypical scrapie isolate inoculated in the same models. Both models challenged with ShTgSPON show the characteristic 7–10 kDa fragment indistinguishable from atypical scrapie, indicating the spontaneous formation of an atypical ovine prion in TgSh112I model. Anti PrP antibody 12B2, 1:5000. MW: Molecular weight

As observed in the ovinized models, the neuropathological and PrP^res^ biochemical features from bank voles (Fig. 8) and both TgVole mouse lines (Fig. 9 and Additional file 1: Fig. S4) were indistinguishable between ShTgSPON and atypical scrapie inocula. In the TgVole models, both inocula induce a severe neuronal loss and gliosis of the hippocampus, particularly involving the CA1 pyramidal cell layer, as well as variable (patchy) loss of granules of the cerebellar granular layer. The thalamus, but not the hypothalamus, the striatum and the neocortex are more severely affected in the overexpressing model TgVole (4x) while in the TgVole (1x) model the lesions and PrP^res^ are almost restricted to the hippocampus and cerebellar cortex (Additional file 1: Fig. S4).

### Lack of transmission to humanized models

To assess the zoonotic potential of our new spontaneous isolate, two transgenic mice expressing human PrP^C^ were inoculated with ShTgSPON: Tg340 (Met129) and Tg361 (Val129). Experimental transmission of atypical scrapie isolates to humanized transgenic mouse models has not been reported so far, at least on first passage [73, 82]. No evidence of transmission to humanised models has been observed: 0/12 Tg340 (Met129) animals (> 959dpi) and 0/10 Tg361 (Val129) (> 840 dpi). Based on these results, the inoculum does not seem to transmit to human PrP^C^ expressing hosts, at least on first passage.

### ShTgSPON is transmissible to sheep and produces the atypical scrapie phenotype

**Fig. 10:**
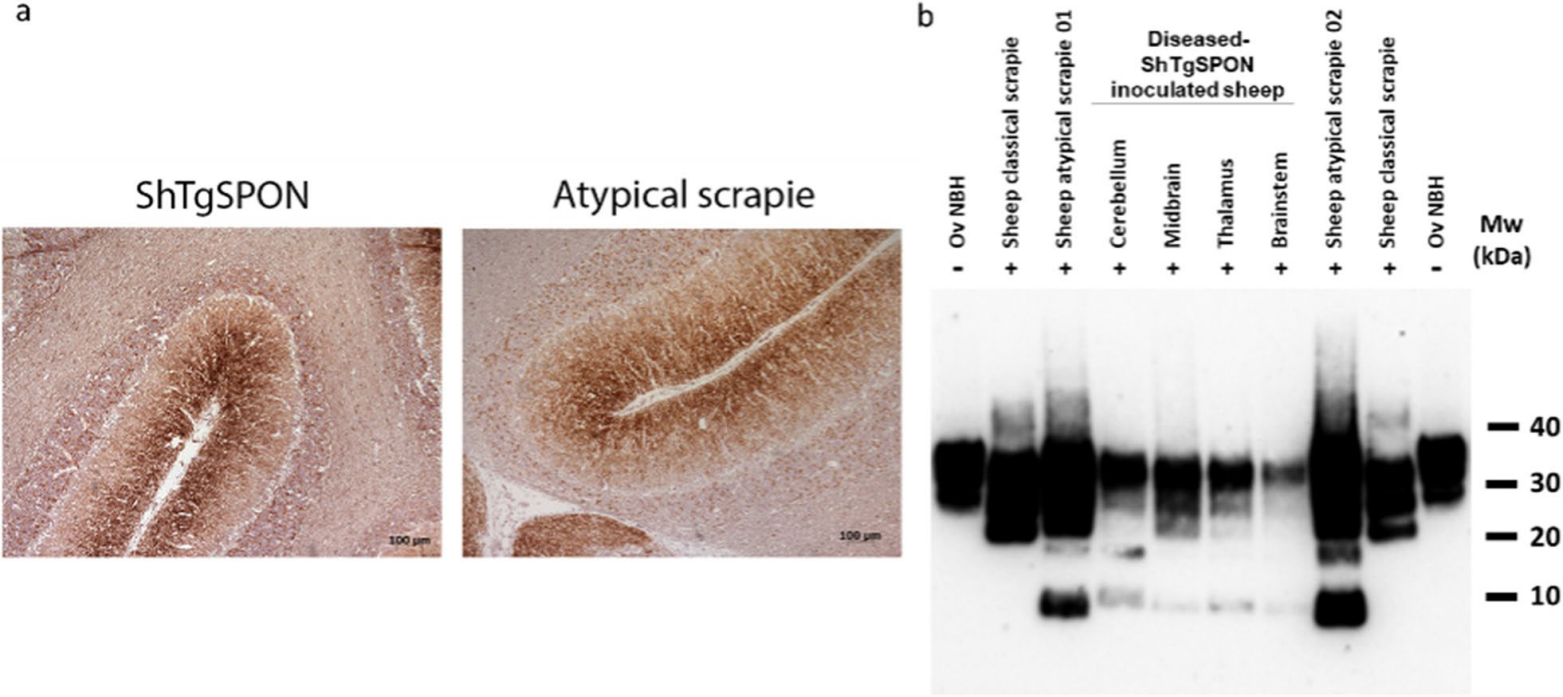
Imunohistochemical and biochemical analysis of sheep inoculated with ShTgSPON compared to atypical scrapie. **a** PrP^res^ Immunohistochemistry showed a fine granular deposition pattern, particularly abundant in the outer molecular layer of the cerebellar cortex that is identical to that observed in sheep inoculated intrecerebrally with atypical scrapie isolate (Anti PrP^res^ antibody 8G8, 1:1000). **b** Western blotting comparison of a healthy sheep brain homogenate (Ov NBH), a classical scrapie isolate (SSBP/1), two different isolates of atypical (Nor98-like) ovine scrapie (01: Cerebellum O-1233 AHQ/AHQ; 02: Cerebellum O-1238 AHQ/AHQ) and the prions extracted from ShTgSPON inoculated AHQ/AHQ sheep after digestion with proteinase K (PK). Anti PrP antibody 12B2, 1:5000. Electrophoretic migration profile of PrP^Sc^ detected in different brain areas of one of the sheep inoculated with diseased-TgShI112 brain homogenate (ShTgSPON), shows a 7–10 kDa band characterisitc of atypical prion disease of sheep, similar to atypical scrapie isolates from sheep used here to compare. Some variability can be observed among the different brain areas regarding the exact size of this low molecular weight band, matching with subtle differences among the two different atypical scrapie isolates used for comparison, likely due to the presence of fragments with ragged ends after PK digestion more evident in low molecular weight products. MW: Molecular weight

The last bioassay proving that a *bona fide* atypical scrapie strain is spontaneously generated in the TgShI112 model was performed in its original host, the sheep. Four sheep of the churra-tensina breed (2 AHQ/AHQ and 2 AHQ/ARR) where intracerebrally inoculated with the ShTgSPON innoculum. One AHQ/AHQ sheep started showing clinical signs compatible with atypical scrapie 18 months post-inoculation (mpi). This animal reached end-point criteria and was euthanized at 30 mpi. The remaining sheep are still alive at the moment of writing this report. Immunohistochemical and biochemical studies showed that the sheep had a disease phenotype identical to that of sheep intracerebrally inoculated with atypical scrapie (Fig. 10).

## Discussion

The following discussion is based on the consideration of prion diseases into two broad categories: acquired (when the source of prions is an external one that seeds the misfolding of the host protein) versus spontaneous (where the misfolding event occurs spontaneously in an individual either idiopathic in nature or favoured by the host genotype, in terms of the presence of a pathogenic mutation on the *PRNP* gene and its expression levels). Modelling prion diseases of genetic or idiopathic spontaneous origin in animal models is highly challenging. In the first case, because mutations associated to genetic human prion disorders in the context of other PrP sequences do not always faithfully mimic the human pathology [30, 43, 74, 84]. Regarding human idiopathic spontaneous prion disorders, in which spontaneous misfolding of PrP^C^ is believed to occur in the absence of mutations, their low incidence (1–5 cases per million inhabitants/year) precludes their systematic investigation in animals with wild type *PRNP* gene and absence of PrP^C^ overexpression. However, given that idiopathic spontaneous prion diseases are the most common in humans, accounting for 80–85% of the cases of transmissible spongiform encephalopathies diagnosed, it is important to develop animal models for such diseases. This lack of models for spontaneous prion diseases was nonetheless partially solved by the finding of bank voles (*Myodes glareolus*) and their unusual behaviour towards prion infection. Evidences have been published that the particular composition of the bank vole PrP^C^, not only renders bank voles highly susceptible to most of all known prion strains [7, 44, 54, 62, 75], but it is also responsible for generating a spontaneous and transmissible prion disease when expressed in a transgenic mouse model [17, 29, 56, 57, 76]. Different amino acid variants particular to bank voles have been suggested to be the drivers of this enhanced susceptibility and proneness to spontaneous misfolding, including D227E and R230S of the C-terminal domain [44] and, particularly, isoleucine at position 109, a residue that is also present in other species [56, 57, 76]. The latter (I109) when overexpressed in a transgenic mouse model spontaneously engenders a transmissible prion disease and the length of its incubation period inversely correlates with the expression levels of the 109I-PrP^C^ [56, 57, 76]. That can explain why, to date, no spontaneous prionopathies have been described in wild type bank voles with this polymorphism. This is the key of the success of the overexpression approach of both the TgVole and our model, in which the presence of this polymorphism is necessary but not sufficient to generate prions spontaneously. It is only upon overexpression that prion disease develops, probably related to endoplasmic reticulum stress and proteasome impairment [56]. A further confirmation of the relevance of the PrP^C^ expression levels is the report of a genetic CJD occurrence in a family in which only the homozygous members developed the disease [83].

The 109I polymorphism was of particular interest in our line of research since the homologous polymorphism in sheep (I112) actually exists in nature, particularly in the Tibetan sheep breed [85]. Other polymorphisms have been described in this locus, such as the T112 which is associated to certain resistance to classical scrapie [33, 42]. Nevertheless, no scrapie cases either classical or atypical have been reported in sheep with the I112 polymorphism, which can probably be explained due to insufficient expression levels and the low frequency of the polymorphism. The aim of our research was to assess whether overexpression of I112 *PRNP* polymorphism in a transgenic mouse model would also elicit the spontaneous generation of a transmissible prion, in order to obtain a laboratory model to study the pathogenesis of prionopathies in ruminants, avoiding exogenous prion administration.

Thus, the TgShI112 transgenic mouse model was generated and, indeed, it spontaneously developed a prion disease with all the neuropathological hallmarks of this group of diseases: neurological clinical signs, spongiform degeneration, astro and microgliosis and PrP^res^ deposition. A certain variability was observed in the incubation period and the penetrance was not a 100%, this is similar to human TSEs associated to *PNRP* mutations in which a wide variability on the age of onset among individuals carrying the same mutation has been observed [51]. Additionally, it was transmissible upon intracerebral inoculation not only to the same model, but also to other models carrying wild type ovine PrP^C^ (Tg338, Tg501 and most interestingly to the original host: AHQ/AHQ sheep) and to models expressing bank vole PrP^C^ (TgVole 1x & 4x, and bank vole). In addition, bioassays on humanized transgenic mice have not shown so far, at least on first passage, a zoonotic potential.

Upon biochemical characterization (Fig. 2) the electrophoretic migration profile of the PrP^res^ deposited in TgShI112 mice brains after proteinase K (PK) digestion strongly resembled the partially PK-susceptible multi-banded patterns with a 7–10 kDa band described in atypical scrapie cases [10]. Some variability was observed in the banding pattern form individual animals that developed disease spontaneously and also in those inoculated with the ShTgSPON inoculum, mainly regarding the presence or absence of a band of approximately 15 kDa (detectable in the sample from Fig. 2, but absent in sample from Fig. 4), whereas high molecular weight bands and the characteristic 7–10 kDa band remain unaltered, which could indicate strain variability. However, the same difference was also observed in the atypical scrapie isolate used as control, suggesting that it might be due to technical variability rather than real strain differences. In fact, freezing and thawing of individual samples seemed to have some effect on the presence or absence of such band and also regarding intensity of high molecular weight bands (data not shown). Moreover, the gel used in Fig. 2 was from a different brand than the rest, what could exacerbate the apparent differences, but since the lowest molecular weight band, the most significant signature of these atypical prions, was unaltered we consider that the spontaneously generated strain was likely conserved upon passage. Taking into account that atypical scrapie is the strain supposedly associated with idiopathic spontaneous prion disease in small ruminants [10], the model presented here exceeds our expectations regarding faithful recreation of a sporadic transmissible spongiform encephalopathy. It provides the opportunity to study systematically the spontaneous development of an atypical and transmissible prion disorder neuropathologically indistinguishable from that found in nature, in an animal model bearing a naturally occurring sheep polymorphic PrP^C^ variant and with expression levels close to physiological. Additionally, TgShI112 mice were readily susceptible to inoculation with an atypical scrapie isolate, demonstrating their usefulness to model acquired atypical prion disease in addition to the spontaneous one. In fact, the resulting lesional pattern was indistinguishable from that obtained when this model was inoculated with the ShTgSPON inoculum (obtained from pooling five brains from TgShI112 with confirmed prion disease). The lesional and PrP^res^ deposition profile observed in TgShI112 mice inoculated with both ShTgSPON and atypical scrapie distinctly involved the cerebellum while sparing the remaining brain areas. This differed considerably from the profile observed in the spontaneously sick ShTgI112 mice in which other areas such as the hippocampus, thalamus, striatum and neocortex were consistently involved. An explanation for this difference could be the length of the incubation period, which in both cases (152 dpi and 177 dpi, respectively) was way shorter than the time required for the spontaneous phenotype to occur; the cerebellum in this model is likely a brain area particularly (and interestingly likewise) susceptible to prions from both isolates upon inoculation. These animals developed a marked ataxia that led to their euthanasia, had the disease been allowed to progress further, it is likely that the prosencephalic brain regions would have been involved and, ultimately would have overlapped with the spontaneous phenotype.

Altogether, these results suggest that this novel animal model gives rise to a prion isolate, the ShTgSPON, with strain features similar to those of Nor98-like atypical scrapie isolates and that it could be the most suitable animal model available not only to investigate the spontaneous misfolding event but also, for any other aspect related to transmission and pathogenesis of atypical prions. Despite the similarities between ShTgSPON and Nor98 regarding biochemical and histopathological features upon inoculation in TgShI112, differences were detected in terms of incubation periods. Such differences may likely respond to distinct titres from the two inocula, although the possibility of being slightly different strains cannot be completely ruled out. The emergence of potentially distinct atypical prion strains in this model, if confirmed, would also be of great interest. Given the low number of atypical prion disease in nature [considering atypical scrapie, some GSS cases and Variably Protease-sensitive Prionopathy (VPSPr) within this category due to the biochemical signature of their PrP^res^], but the great phenotypical heterogeneity among them [26, 50, 60, 63, 71], a largely uncharacterized strain variability could exist which could be assessed through this new model.

To further ascertain this finding, multiple bioassays were carried out in transgenic mouse models and in sheep to compare the ShTgSPON isolate with an ovine atypical scrapie isolate. The results obtained in all the challenged models (Tg338, Tg501, TgVole, bank vole and AHQ/AHQ sheep) showed strikingly similar neuropathological and biochemical features, thus confirming our initial suspicion. Along this line, both inocula showed little transmission barrier when inoculated in the Tg338 model as shown by the slight shortening of the incubation period on the second passage (Table 2). Most interestingly, the preliminary results of the sheep bioassay (Fig. 10) definitely show that the phenotype encoded in ShTgSPON inoculum is undistinguishable from that generated with an intracerebral inoculation of atypical scrapie isolate.

Remarkably, transmission studies of atypical prions to either wild type animals, or models bearing isoleucine in the position equivalent to that of bank voles, showed an enhanced susceptibility to ShTgSPON and natural atypical scrapie isolates. TgShI112 models, despite their lower levels of expression when compared to Tg338 (8x) and Tg501 (2x), showed generally much shorter incubation periods upon inoculation of ShTgSPON and atypical scrapie isolates. Similarly, bank vole-based models (bank voles with I109 polymorphism, TgVole 1 × and 4x), show 100% attack rates and strikingly short incubation periods. While bank voles with M109 polymorphism do not succumb to infection with atypical scrapie isolates in more than 600 days [61]. This indicates that isoleucine at position 109 from bank vole or equivalents could favour propagation of atypical forms of prion disease in a generalized manner, independently from transmission barriers, what may help gain insight on the differences between classical and atypical prion strains.

Even though the I112 polymorphism, upon overexpression, favours misfolding of ovine PrP^C^ with atypical scrapie strain features, it is obviously not the sole determinant of the spontaneous appearance of Nor98 strain in sheep, since no atypical scrapie cases have been described among animals bearing this polymorphism. Although the low number of sheep with such variant and the absence of active scrapie surveillance in Asia may have precluded detection. Atypical scrapie has not been linked to a particular *PRNP* mutation, but the presence of phenylalanine in codon 141 [53] and the presence of a histidine in codon 154 [21] have been associated with this phenotype, however, these polymorphisms neither do account for all cases.

While TgShI112 mice were apparently more susceptible to atypical scrapie than the wild type counterparts were, they showed very low susceptibility to one strain causing classical scrapie (SSBP/1). Nonetheless, the fact that one of the inoculated animals developed disease, and that inoculation with sheepBSE resulted in a 100% attack rate with a distinguishable lesion profile, undoubtedly demonstrate that classical prion disease can also be modelled in this transgenic mouse line. A possible explanation to the low susceptibility of these models to the classical scrapie strain used herein may come from other polymorphic positions within ovine PrP. The genotype of the TgShI112 animals is ARQ (A136, R154 and Q171) regarding the three most studied polymorphic sites in sheep PrP^C^ influencing susceptibility to scrapie, which has been already reported to show lower susceptibility than other genotypes to SSBP/1 inoculation [39]. Along this line, analysing the effect of alternative genotypes together with I112 polymorphism on the propagation of classical and atypical scrapie strains could be of great interest to determine whether some other amino acid variant could hinder formation and propagation of one strain type versus the other. However, as it is probably the case for 141F and 154H, the PrP^C^ bearing the I112 polymorphism seems to be more prone to misfold into a structure encoding the atypical scrapie strain rather than other polymorphisms that favour the classical counterparts. Accordingly, atypical cases are frequently found in classical scrapie-resistant genotypes [24, 28, 45, 65].

In summary, we have herein presented experimental evidence to claim that expression of the sheep PrP^C^ with an isoleucine in position 112 in a transgenic mouse model, results in the spontaneous generation of a *bona fide* transmissible prion disease with strain features undistinguishable from small ruminants atypical/Nor98 scrapie. This model will be of great interest on the one hand, to study the pathogenesis of atypical sporadic prion phenotypes in small ruminants, such as where does the initial misfolding event take place or how long before disease onset is PrP^res^ detectable and in which tissues. On the other hand, it may also be an invaluable model to test anti-prion therapeutic agents as a model of human prionopathies with atypical PrP^res^ features such as GSS and VPSPr.

## Supplementary Information

**Additional file 1**. Supplementary figures 1 to 4. **Fig. S1** PrP^C^ expression levels in TgShI112 mouse lines L456 and L460 compared to a normal sheep brain PrP^C^ expression by Western blotting. **Fig. S2** Anatomopathological analysis of sheepBSE-inoculated TgShI112 mice. **Fig. S3**: Comparative anatomopathological analysis of Tg338 (Ovine VRQ PrP^C^) and Tg501 (ovine ARQ PrP^C^) mice inoculated with TgShSPON, atypical scrapie and SSBP/1. **Fig. S4**: Analysis of ShTgSPON-inoculated compared to atypical scrapie-inoculated TgVole mice.

## Data Availability

The datasets during and/or analysed during the current study available from the corresponding author on reasonable request.
