# Supplementary material for: Bona fide atypical scrapie faithfully reproduced for the first time in a rodent model
Source: Acta Neuropathol Commun. 2022 Dec 13;10:179. doi: 10.1186/s40478-022-01477-7 (PMC9749341; doi:10.1186/s40478-022-01477-7)
Supplement: Supplementary file 1 — Additional file 1. Supplementary figures 1 to 4. Fig. S1 PrPC expression levels in TgShI112 mouse lines L456 and L460 compared to a normal sheep brain PrPC expression by Western blotting. Fig. S2 Anatomopathological analysis of sheepBSE-inoculated TgShI112 mice. Fig. S3: Comparative anatomopathological analysis of Tg338 (Ovine VRQ PrPC) and Tg501 (ovine ARQ PrPC) mice inoculated with TgShSPON, atypical scrapie and SSBP/1. Fig. S4: Analysis of ShTgSPON-inoculated compared to atypical scrapie-inoculated TgVole mice. [file 40478_2022_1477_MOESM1_ESM.docx]

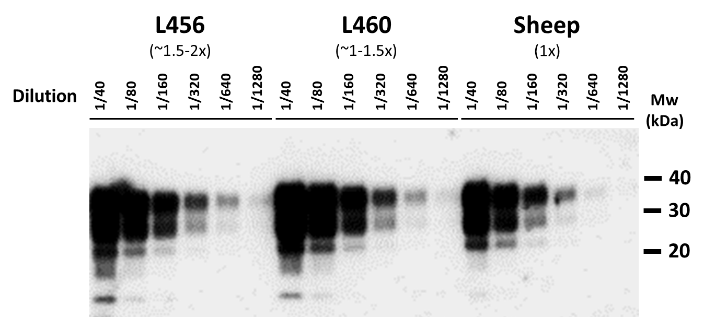


**Fig. S1** **PrP^C^ expression levels in TgShI112 mouse lines L456 and L460 compared to a normal sheep brain PrP^C^ expression by Western blotting**. 10% brain homogenates from both mouse lines and sheep were diluted 1:40, 1:80, 1:160, 1:320, 1:640 and 1:1280 and analyzed by Western blot using monoclonal antibody D18 (1:5000). The PrP^C^ expression levels L456 and L460 were approximately 1.5-2x and 1-1.5x, respectively compared to PrP^C^ levels in sheep brain, based on signal intensity. Notice that the glycosylation pattern is maintained between the transgenic mice lines and the sheep showing a correct posttranslational processing of the PrP^C^ in both mouse lines. Mw: Molecular weight.


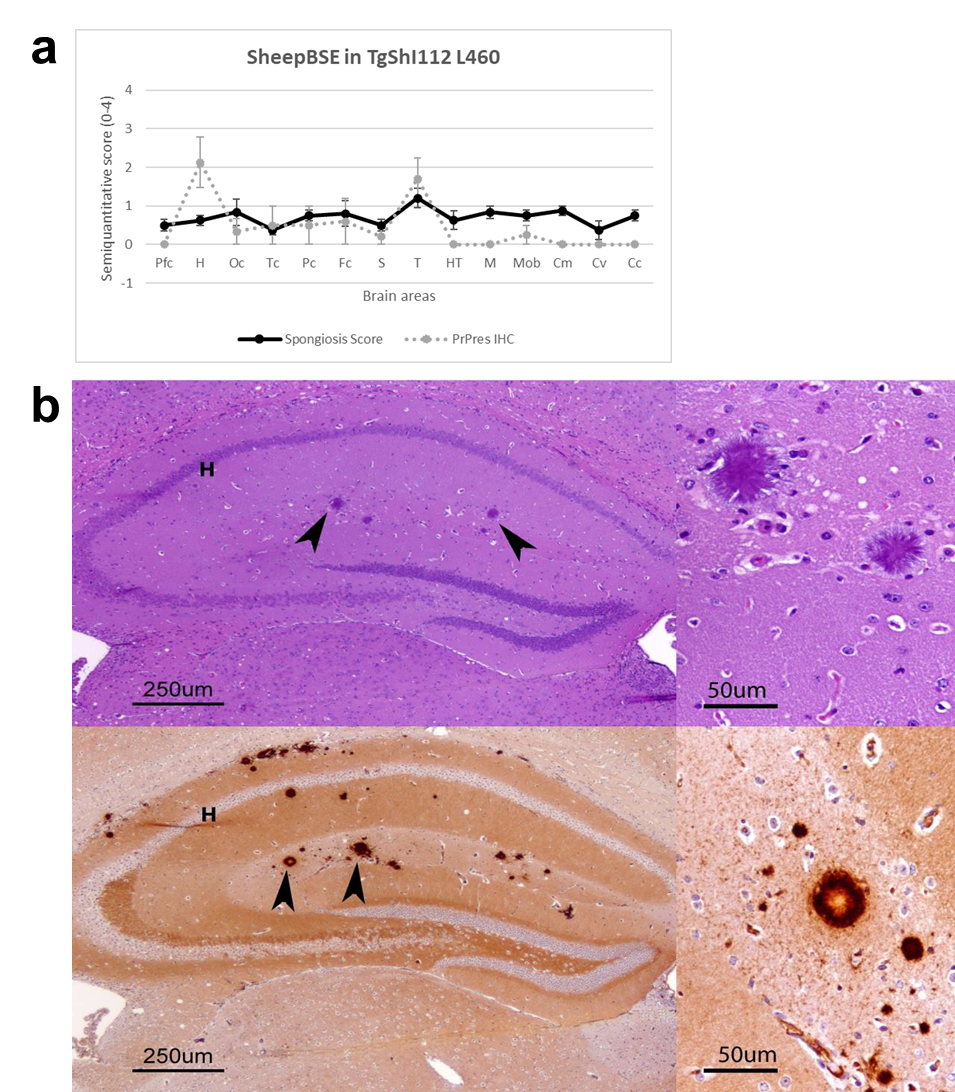


**Fig. S2 Anatomopathological analysis of sheepBSE-inoculated TgShI112 mice. (a) Brain lesion and PrP^res^ deposit distribution for the inoculum sheepBSE in TgShI112 mice (n=5)**. Brain lesion profiles and PrP^res^ deposition profiles represent the mean semi-quantitative scoring (0–4, vertical axis) of the spongiform lesions (continuous line) and the immunohistochemical labelling of PrP^res^ deposits (dashed line) against 14 brain regions (Pfc: piriform cortex, H: hippocampus, Oc: occipital cortex, Tc: temporal cortex, Pc: parietal cortex, Fc: frontal cortex, S: striatum, T: thalamus, HT: hypothalamus, M: mesencephalon, Mob: medulla oblongata, Cm: cerebellar nuclei, Cv: cerebellar vermis, Cc: cerebellar cortex). Bars: standard error of the mean. (**b**) **Neuropathological characterisation of the lesions** (H&E staining, top) and PrP^res^ immunohistochemistry (2G11, 1:100 bottom) in the brains TgShI112 mica inoculated with sheepBSE. Notice the plaque-like rounded extracellular PrP^res^ deposits.


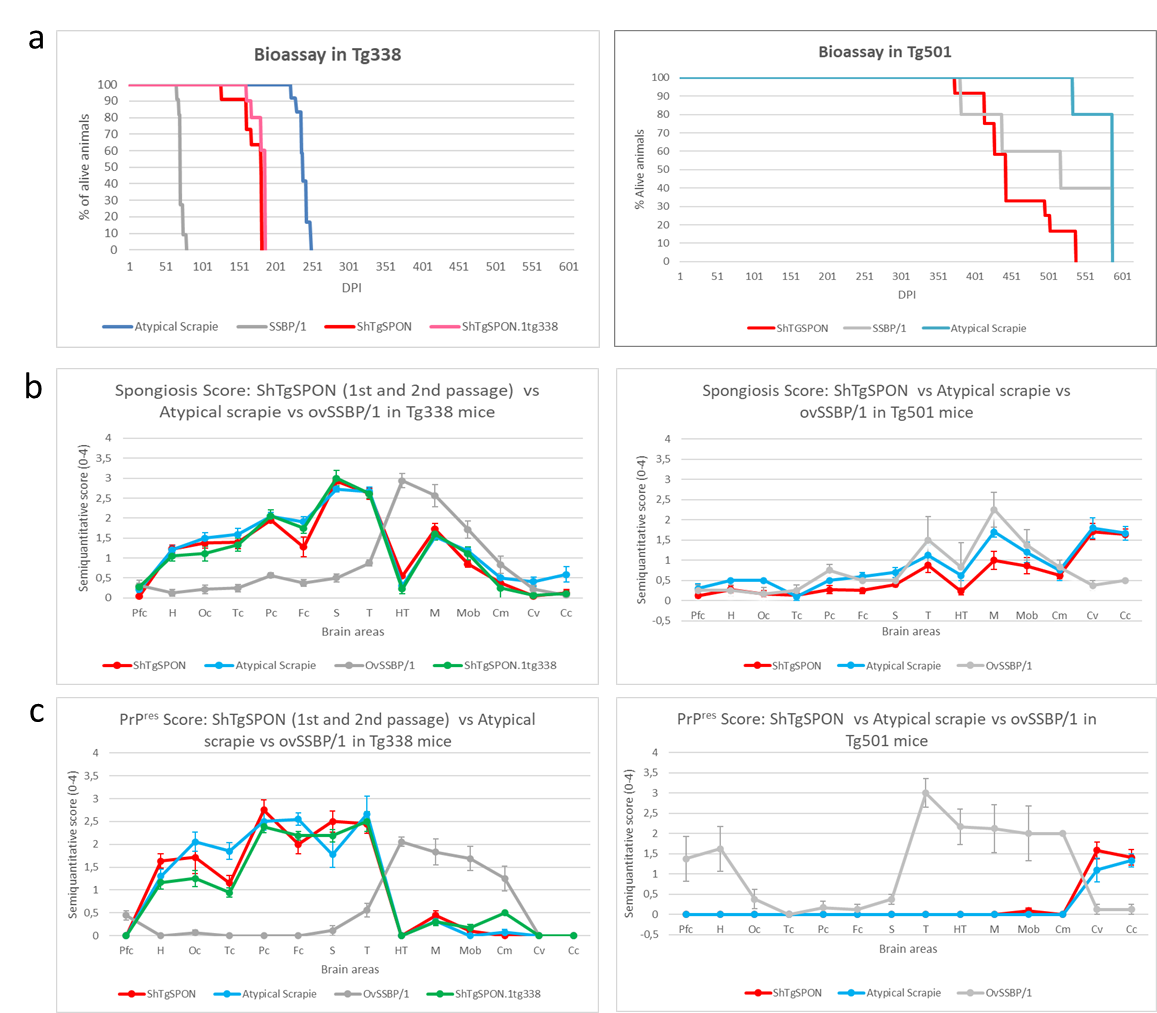


**Fig. S3**: **Comparative anatomopathological analysis of Tg338 (Ovine VRQ PrP^C^) and Tg501 (ovine ARQ PrP^C^) mice inoculated with TgShSPON, atypical scrapie and SSBP/1**. **(a)** **Kaplan-Meyer survival curves** comparing ShTgSPON (1^st^ passage red, 2^nd^ passage (green) inocula, an atypical scrapie isolate (blue) and a classical scrapie isolate (SSBP/1 grey) incubation periods in the tg338 and Tg501 mouse models. Notice the lack of adaptation of the ShTgSPON inoculum upon second passage, indicating an absence of transmission barrier. The classical scrapie isolate is roughly 100 days faster than the ShTgSPON isolate in both models. **(b) Brain lesion and (c) PrP^res^ deposit distribution for the inoculum ShTgSPON (1^st^ passage red and 2^nd^ passage pink for Tg338 only), atypical scrapie (blue) and classical scrapie (SSBP/1, grey) in Tg338 (n=11, 10, 12 and 11 respectively) and Tg501 (n=12, 5 and 4, respectively) mice.** Brain lesion profiles and PrP^res^ deposition profiles represent the mean semi-quantitative scoring (0–4, vertical axis) of the spongiform lesions (continuous line, blue) and the immunohistochemical labelling of PrP^res^ deposits (dashed line, blue) against 14 brain regions (Pfc: piriform cortex, H: hippocampus, Oc: occipital cortex, Tc: temporal cortex, Pc: parietal cortex, Fc: frontal cortex, S: striatum, T: thalamus, HT: hypothalamus, M: mesencephalon, Mob: medulla oblongata, Cm: cerebellar nuclei, Cv: cerebellar vermis, Cc: cerebellar cortex). Notice the striking similarity of the brain profiles obtained with atypical scrapie and ShTgSPON isolates and their remarkable differences with the classical SSBP/1 isolate. Bars: standard error of the mean,


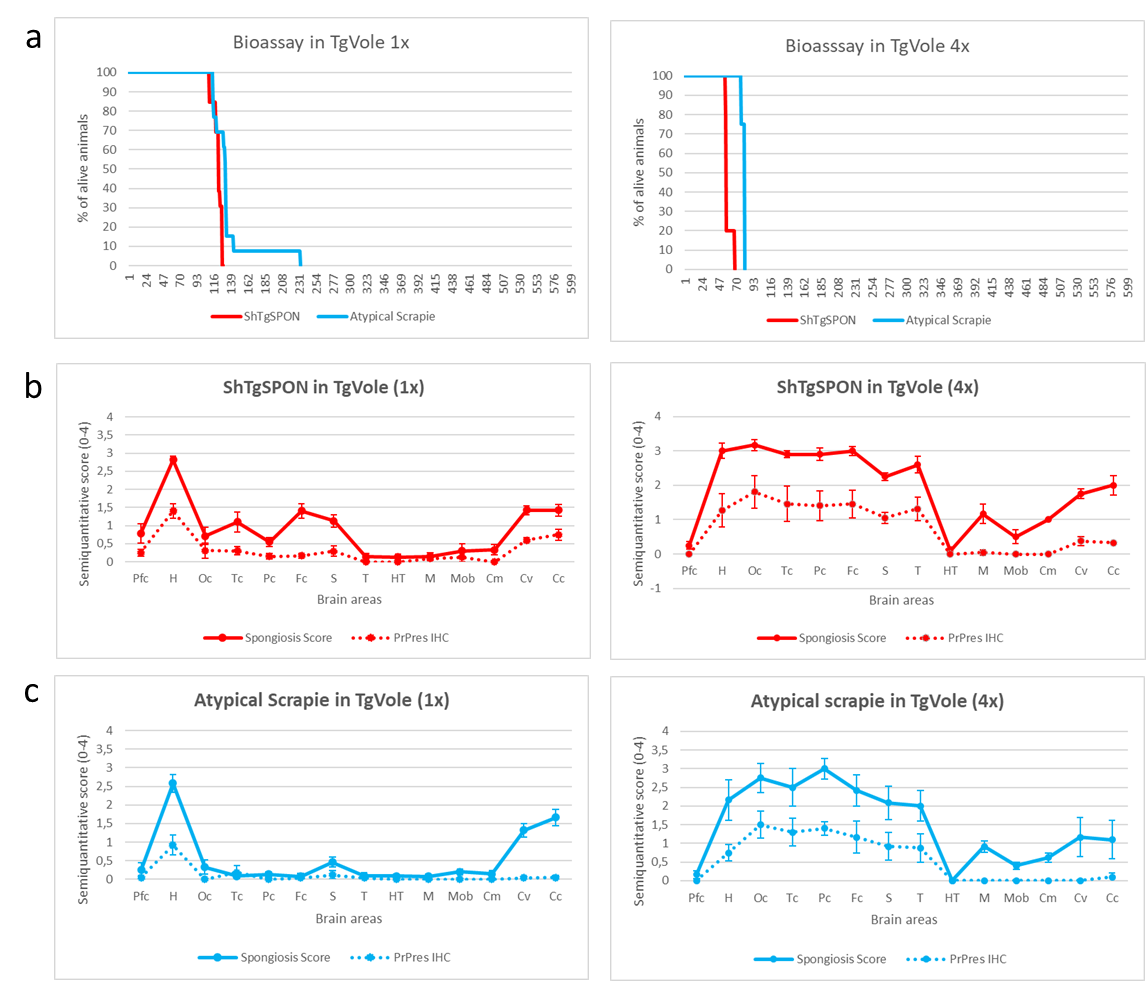


**Fig. S4: Analysis of ShTgSPON-inoculated compared to atypical scrapie-inoculated TgVole mice. (a) Kaplan-Meyer survival curves** comparing ShTgSPON (red) inoculum and an atypical scrapie isolate (blue) incubation periods in the TgVole(1x) and TgVole(4x). Notice the strikingly reduced incubation period in the TgVole (4x) mice. **(b) Brain lesion and PrP^res^ deposit distribution for the inoculum ShTgSPON and (c) atypical scrapie in TgVole (1x) (n=13 and 13) and TgVole (4x) (n=6 and 6) mice.** Brain lesion profiles and PrP^res^ deposition profiles represent the mean semi-quantitative scoring (0–4, vertical axis) of the spongiform lesions (continuous line, blue) and the immunohistochemical labelling of PrPres deposits (dashed line, blue) against 14 brain regions (Pfc: piriform cortex, H: hippocampus, Oc: occipital cortex, Tc: temporal cortex, Pc: parietal cortex, Fc: frontal cortex, S: striatum, T: thalamus, HT: hypothalamus, M: mesencephalon, Mob: medulla oblongata, Cm: cerebellar nuclei, Cv: cerebellar vermis, Cc: cerebellar cortex). Notice the striking similarity of the brain profiles obtained with both isolates. Bars: standard error of the mean.
